# Associations between nine dietary minerals intake and all-cause mortality in individuals with atherosclerotic cardiovascular disease

**DOI:** 10.3389/fnut.2024.1447167

**Published:** 2024-10-14

**Authors:** Chenglin Duan, Meng Lv, Xintian Shou, Zizhen Chen, Yujie Luan, Yuanhui Hu

**Affiliations:** ^1^Department of Cardiovascular, Guang’anmen Hospital, China Academy of Chinese Medical Sciences, Beijing, China; ^2^Graduate School, Beijing University of Chinese Medicine, Beijing, China; ^3^Department of Cardiovascular, Xiyuan Hospital, China Academy of Chinese Medical Sciences, Beijing, China

**Keywords:** dietary minerals intake, atherosclerotic cardiovascular disease, mortality, body mass index, stratified analyses

## Abstract

**Background:**

Varied intake of dietary minerals critically affects cardiovascular health. This study examines the associations of nine dietary minerals intake with all-cause mortality in atherosclerotic cardiovascular diseases (ASCVDs).

**Methods:**

This study analyzed 4,125 individuals with ASCVD from the National Health and Nutrition Examination Survey, employing Kaplan–Meier survival analyses, weighted Cox models, and restricted cubic splines to assess linear and nonlinear relationships between dietary minerals intake and all-cause mortality. Associations across different body mass index (BMI) categories were also evaluated separately.

**Results:**

Over 6.25 years of median follow-up, 1,582 deaths were documented. Adjusted for potential covariates, results show a negative linear correlation between dietary magnesium intake and all-cause mortality (*p* for trend <0.001). Compared to the lowest quartile, all-cause mortality risk in the highest quartile was found to be 0.63 (95% CI 0.49–0.81). The associations between intake of the other eight dietary minerals and all-cause mortality were not robust. BMI significantly influenced the links between dietary minerals intake and all-cause mortality (*p* for interaction <0.05). Across BMI categories, significant negative associations were found between intake of magnesium, phosphorus, potassium, sodium, and copper and all-cause mortality in underweight or normal weight groups. In overweight individuals, intake of calcium, iron, magnesium, and potassium was negatively linked to all-cause mortality. For obese groups, sodium intake negatively affected all-cause mortality (*p* for trend <0.001).

**Conclusion:**

Unlike other dietary minerals, increased magnesium intake significantly reduced all-cause mortality risk in ASCVD. BMI influenced the associations between dietary minerals intake and all-cause mortality.

## 1 Introduction

Atherosclerotic cardiovascular disease (ASCVD) remains a principal factor in morbidity and mortality all around the world, markedly affecting the global public challenge of disease (1). According to guidelines, ASCVD encompasses a range of conditions, including coronary artery disease, stroke, and peripheral artery disease, primarily induced by the accumulation of plaques within arterial walls, leading to chronic ischemia and hypoxia in tissues (2). Despite considerable progress in therapeutic interventions and preventive measures, the occurrence and associated mortality of ASCVD persist as a significant challenge to public health. Identifying modifiable factors to prevent or mitigate premature mortality in individuals with ASCVD is crucial. Effective dietary management, emphasizing particular nutritional components, is critical in influencing the onset and advancement of ASCVD. A series of studies highlights the considerable impact of nutritional factors on cardiovascular outcomes (3, 4).

The intake of dietary minerals is essential in cardiovascular outcomes, with ongoing interest in the potential of these nutrients to enhance the prognosis of individuals with ASCVD. Recent epidemiological research in populations and foundational experiments in animals have uncovered links between minerals and ASCVD. Dietary minerals such as zinc, magnesium, iron, and copper have been identified as having potential effects on the occurrence and prognosis of ASCVD (3–5). Iron, zinc, copper, and selenium, key transition metals, are crucial in cellular metabolism, significantly influencing atherosclerosis through their oxidative and antioxidative properties (6–9). Iron overload accelerates atherosclerosis development through elevation of pro-inflammatory mediators, impairment of endothelial function, and mobilization of immune cells (5). Zinc mitigates atherosclerosis progression by altering low-density lipoprotein oxidation and preserving the health of vascular endothelial and smooth muscle cells (10, 11). Cu impacts atherosclerosis by modulating lipid metabolism, oxidative stress, and inflammatory pathways involved in plaque formation (7, 12). Selenium plays a preventative role against atherosclerosis associated with cardiovascular disease by protecting cells from excessive reactive oxygen species, mitigating oxidative stress, regulating inflammation, preventing endothelial dysfunction, minimizing lipid peroxidation, and safeguarding against apoptosis (13, 14). Zinc, through its reduction of C-reactive protein, pro-inflammatory cytokines, cell adhesion molecules, and markers of oxidative stress, demonstrates potential in the prevention and slowing of atherosclerosis progression, attributed to its anti-inflammatory and antioxidative properties (15). A prospective study within an Asian cohort revealed an inverse relationship between the amount of magnesium ingested from food and mortality due to ASCVD (16). Another prospective cohort study identified an inverse association between the amount of zinc ingested from food and mortality from coronary heart disease in males (3).

Despite knowledge of dietary minerals’ potential benefits or harms for individuals with ASCVD, large-scale prospective research on their association with all-cause mortality in the group of individuals with ASCVD remains scarce. This investigation aims to address the existing by analyzing data from the National Health and Nutrition Examination Survey (NHANES), with a focus on the relationship between specific mineral intake and the mortality risk in individuals with ASCVD. It intends to provide evidence that may inform dietary guidelines and interventions designed to alleviate the burden of atherosclerotic cardiovascular diseases. Our hypothesis suggests that in individuals with ASCVD, increased intake of certain minerals correlates with a decrease in all-cause mortality from ASCVD, with this relationship potentially modified by specific factors.

## 2 Materials and methods

### 2.1 Data collection and study participants

The NHANES is a countrywide investigation designed to assess relationships between nutritional health and health outcomes in adult and pediatric populations. NHANES recruited samples of the civilian noninstitutionalized US population using a multistage probability sampling method in continuous two-year cycles. The National Center for Health Statistics and Ethics Review Board approved the protocol for NHANES, and all participants provided written informed consent. This prospective cohort study included 80,312 participants from eight NHANES survey cycles (2003–2018). The self-reported health status questionnaire identified 4,702 individuals with ASCVD. After excluding individuals with incomplete data on mortality (*n* = 8) or dietary minerals intake (*n* = 569), the final analyses included 4,125 individuals diagnosed with ASCVD. Details on participant recruitment are depicted in Supplementary Figure S1.

### 2.2 Measurement of dietary minerals

NHANES applied a bias-minimized retrospective dietary assessment approach to quantify dietary minerals intake. Dietary minerals intake information was gathered from detailed 24-h dietary reminisce provided by participants. Dietary minerals intake data were estimated by aggregating the mineral content of each food and beverage consumed 24 h prior to the interview. Daily intakes of dietary nutrients from all foods and beverages were computed utilizing the US Department of Agriculture Food and Nutrient Database for Dietary Studies, connected with the NHANES database. The NHANES interview and examination procedures manual contains detailed structured dietary interview questionnaires.

### 2.3 Definition of atherosclerotic cardiovascular diseases and mortality outcome

According to the 2013 ACC/AHA Guideline, ASCVD is identified by possessing one or more of the following conditions: coronary heart disease, angina, heart attack, or stroke (2). Self-reported ASCVD was ascertained using structured health status questionnaires by NHANES personnel, with relevant data accessible via codes “mcq160c,” “mcq160d,” “mcq160e,” “spq070e,” “mcq160f,” and “spq070d” in the NHANES database. To ascertain the participants’ mortality status, mortality follow-up time was obtained from the National Death Index records, updated as of December 31, 2019, and this information was linked to the NHANES database. All-cause mortality was characterized as demise resulting from any reason. The duration of follow-up was quantified in months, commencing from the date of the household dietary recall interview and concluding on the earlier of two events: the date of passing or the termination of the follow-up period on December 31, 2019.

### 2.4 Covariates

Potential covariates were determined from previously published studies and clinical plausibility (17–19). This study included key demographic characteristics: age, sex (male/female), race/ethnicity (non-Hispanic Black, non-Hispanic White, Mexican American, other), and educational attainment (less than high school, high school, college, or above). The study factored smoking status, drinking status, body mass index (BMI), recreational physical activity, and total caloric intake into its analysis as potential covariates. Drinking status was assessed by evaluating lifetime intake of more than 12 drinks and current drinking patterns, leading to classification as non-drinkers, former drinkers, or current drinkers. Smoking status was determined based on participants’ lifetime consumption of at least 100 cigarettes and their current smoking status, categorizing them as non-smokers, former smokers, or current smokers. Recreational physical activity was divided into two levels. Participant energy intake levels were quantified using data obtained from dietary recall interviews. Acknowledging Considering associations between lipid profiles and ASCVD, and the substantial amount of missing data (50%) on low-density lipoprotein and triglycerides, total cholesterol and high-density lipoprotein levels were selected as key laboratory parameters for this analysis. In the study, hyperlipidemia, hypertension, and diabetes were incorporated as covariates in the analysis of patient histories. Diagnoses of hyperlipidemia and diabetes were based on patient self-reports, previous professional diagnoses, and laboratory parameters. History of hypertension were ascertained using self-reported physician diagnoses and blood pressure measurements obtained from the Mobile Examination Center. Utilization of antihypertensive and antihyperlipidemic pharmaceuticals was a significant determinant of outcomes. According to previous reports, the use of antihypertensive and lipid-lowering medications significantly impacts outcomes.

### 2.5 Statistical analysis

Adhering to NHANES guidelines, this study’s analyses applied sample weights, ensuring rigorous and representative results. Participant’s characteristics were detailed, with categorical variables expressed in terms of unweighted frequencies and corresponding weighted percentages. Continuous variables that conformed to a normal distribution were represented by sample weighted means ± standard error (SE), while those not adhering to a normal distribution were depicted by the median (interquartile range). To mitigate the impact of missing covariate data on sample size, we implemented multiple imputation methods with a predefined random seed.

To assess correlations between the amount of minerals ingested from food and all-cause mortality in ASCVD, Kaplan–Meier survival analysis and log-rank tests were utilized. Concurrently, three Cox proportional hazards regression models and trend tests were formulated. Model 1 was preserved without adjustments; Model 2 adjusted for demographic variables (age, sex, race/ethnicity, and education); expanding upon Model 2, Model 3 included adjustments for health behaviors (smoking condition, drinking condition, BMI, recreational physical activity, and total caloric intake), lipid levels (total cholesterol and high-density lipoprotein), historical medical conditions (hyperlipidemia, hypertension, and diabetes), and drug usage (antihypertensive and lipid-lowering drugs).

Stratified analyses by age, sex, BMI, hypertension, and diabetes were further conducted, with a focus on statistically evaluating the interactions between dietary minerals intake and these stratification factors. To explore the nonlinear relationship, weighted restricted cubic splines (RCS) were employed.

Several sensitivity analyses were employed to assess the robustness of the results. (1) To mitigate the risk of reverse causation bias, individuals who occurred events within the initial two-year period of follow-up were omitted from the study. (2) Analyses were conducted on data split into five groups according to quintiles of the amount of minerals ingested from food. (3) To explore the potential effects of liver function on any observed associations, we further adjusted for liver function parameters.

The R software (version 4.3.0) was utilized for the execution of statistical analyses. Statistical significance was established at a two-tailed *p*-value threshold of 0.05. The specific R packages utilized in this study are detailed in the Supplementary methods section.

## 3 Results

### 3.1 Baseline characteristics of study participants

In this study, we included 4,125 individuals with ASCVD, with a median age of 67 years and a majority of 54.66% being male. We have stratified the initial characteristics of the subjects built on their dietary magnesium intake, as summarized in Table 1. The median (interquartile range) dietary consumption was 248.00 (174.00, 337.00) mg for magnesium, 741.00 (465.00, 1,094.00) mg for calcium, 12.29 (8.66, 17.60) mg for iron, 1115.00 (791.00, 1,515.00) mg for phosphorus, 2,387.00 (1,680.00, 3,195.00) mg for potassium, 2,848.00 (2,019.00, 3,900.00) mg for sodium, 9.05 (6.19, 13.54) mg for zinc, 1.02 (0.72, 1.42) mg for copper, and 89.40 (60.80, 126.70) mcg for selenium. Participants in the fourth quartile of dietary magnesium consumption, in comparison to those in the first quartile, were characterized by a younger demographic, a predominance of males, a higher proportion of non-Hispanic White ethnicity, and elevated levels of education. Additionally, these individuals were more likely to report higher dietary energy intakes, engage in more leisure-time physical activity, have lower rates of smoking, and exhibit lower lipid levels. It was also observed that these participants were more inclined to use lipid-lowering medications and less apt to have diabetes in their medical history.

**Table 1:** Baseline characteristics of study participants stratified by quartiles of dietary magnesium intake.

Characteristics		Quartile 1	Quartile 2	Quartile 3	Quartile 4	*p-*value
	Total	(4, 166)	(166, 235)	(235, 324)	(324, 1,704)	
Demographic variables						
Age, years	67 (57, 76)	66 (55, 77)	67 (58, 77)	68 (58, 76)	65 (56, 74)	0.01
Sex (n, %)						< 0.0001
Male	2,380 (54.66)	443 (35.74)	542 (46.20)	629 (56.55)	766 (75.00)	
Female	1,745 (45.34)	602 (64.26)	479 (53.80)	399 (43.45)	265 (25.00)	
Race/ethnicity (n, %)						< 0.0001
Non-Hispanic White	2,311 (74.67)	514 (69.91)	568 (73.07)	631 (78.29)	598 (76.54)	
Non-Hispanic Black	842 (11.10)	300 (16.53)	220 (12.20)	161 (8.74)	161 (7.98)	
Mexican American	442 (4.73)	109 (4.61)	104 (4.74)	109 (4.82)	120 (4.73)	
Other	530 (9.50)	122 (8.94)	129 (9.99)	127 (8.15)	152 (10.75)	
Education (n, %)						< 0.0001
Less than high school	670 (9.61)	220 (14.09)	174 (11.54)	146 (7.80)	130 (6.05)	
High school	1,773 (43.14)	471 (47.77)	476 (48.94)	445 (43.27)	381 (34.59)	
College or above	1,682 (47.25)	354 (38.13)	371 (39.52)	437 (48.93)	520 (59.35)	
Healthy behavioral risk factors						
Smoking status (n, %)						0.01
Non-smokers	1,577 (37.91)	413 (38.83)	405 (39.10)	399 (38.83)	360 (35.39)	
Former smokers	1,677 (39.37)	370 (33.53)	408 (38.21)	445 (41.31)	454 (43.26)	
Current smokers	871 (22.72)	262 (27.64)	208 (22.69)	184 (19.87)	217 (21.34)	
Drinking status (n, %)						< 0.0001
Non-drinkers	575 (12.34)	201 (19.01)	152 (14.17)	128 (10.91)	94 (6.78)	
Former drinkers	1,305 (28.30)	362 (33.83)	362 (32.95)	303 (24.35)	278 (23.61)	
Current drinkers	2,245 (59.37)	482 (47.15)	507 (52.88)	597 (64.74)	659 (69.60)	
Leisure-time physical activity (n, %)						< 0.0001
No	2,989 (68.24)	840 (81.17)	776 (71.66)	722 (64.04)	651 (58.87)	
Yes	1,136 (31.76)	205 (18.83)	245 (28.34)	306 (35.96)	380 (41.13)	
BMI (kg/m^2^)	29.27 (25.60, 33.78)	29.68 (25.76, 34.24)	29.70 (25.52, 33.80)	29.15 (25.62, 33.80)	28.86 (25.57, 33.10)	0.39
Energy intake (kcal)	1,758 (1,302, 2,321)	1,087 (821, 1,420)	1,510 (1,233, 1,856)	1,945 (1,590, 2,330)	2,467 (1,971, 3,160)	< 0.0001
Laboratory parameters						
Total cholesterol (mmol/L)	4.60 (3.93, 5.38)	4.76 (4.06, 5.59)	4.60 (3.90, 5.38)	4.68 (3.96, 5.33)	4.45 (3.83, 5.28)	< 0.001
High density lipoprotein (mmol/L)	1.24 (1.03, 1.53)	1.22 (1.01, 1.50)	1.27 (1.06, 1.55)	1.29 (1.03, 1.55)	1.22 (1.01, 1.50)	0.02
Drug usage						
Antihypertensive drug (n, %)						0.67
No	885 (24.01)	232 (23.49)	203 (23.99)	209 (22.54)	241 (25.74)	
Yes	3,240 (75.99)	813 (76.51)	818 (76.01)	819 (77.46)	790 (74.26)	
Antihyperlipidemic drug (n, %)						0.003
No	1,730 (40.54)	504 (48.62)	400 (38.11)	410 (38.67)	416 (37.72)	
Yes	2,395 (59.46)	541 (51.38)	621 (61.89)	618 (61.33)	615 (62.28)	
Chronic diseases						
Diabetes (n, %)						0.01
No	2,442 (62.59)	596 (58.85)	563 (59.37)	632 (63.84)	651 (67.08)	
Yes	1,683 (37.41)	449 (41.15)	458 (40.63)	396 (36.16)	380 (32.92)	
Hypertension (n, %)						0.12
No	894 (25.04)	195 (21.69)	205 (23.10)	246 (27.76)	248 (26.87)	
Yes	3,231 (74.96)	850 (78.31)	816 (76.90)	782 (72.24)	783 (73.13)	
Hyperlipidemia (n, %)						0.66
No	569 (12.54)	151 (12.84)	147 (13.86)	123 (11.35)	148 (12.27)	
Yes	3,556 (87.46)	894 (87.16)	874 (86.14)	905 (88.65)	883 (87.73)	
Dietary minerals intake						
Calcium (mg)	741 (465, 1,094)	380 (244, 562)	630 (444, 822)	845 (606, 1,132)	1,118 (827, 1,551)	< 0.0001
Iron (mg)	12.29 (8.66, 17.60)	7.42 (5.00, 9.83)	10.35 (8.10, 13.02)	13.81 (10.86, 18.15)	18.30 (14.22, 25.16)	< 0.0001
Phosphorus (mg)	1,115 (791, 1,515)	629 (463, 791)	927 (773, 1,116)	1,239 (1,029, 1,470)	1,688 (1,410, 2,157)	< 0.0001
Potassium (mg)	2,387 (1,680, 3,195)	1,272 (969, 1,587)	2,027 (1,697, 2,360)	2,679 (2,313, 3,086)	3,635 (3,074, 4,324)	< 0.0001
Sodium (mg)	2,848 (2,019, 3,900)	1,869 (1,298, 2,480)	2,475 (1,825, 3,237)	3,207 (2,436, 4,068)	3,862 (3,006, 5,235)	< 0.0001
Zinc (mg)	9.05 (6.19, 13.54)	5.07(3.45, 6.97)	7.67 (5.67, 9.65)	10.10 (7.86, 13.50)	14.41 (10.87, 19.00)	< 0.0001
Copper (mg)	1.02 (0.72, 1.42)	0.56 (0.43, 0.68)	0.85 (0.72, 1.02)	1.14 (0.96, 1.33)	1.62 (1.38, 1.99)	< 0.0001
Selenium (mcg)	89.40 (60.80, 126.70)	55.30 (38.40, 75.10)	76.60 (57.40, 98.90)	99.50 (75.70, 129.10)	134.90 (104.20, 172.60)	< 0.0001

BMI, body mass index.

### 3.2 Kaplan–Meier survival analyses

**Figure 1:**
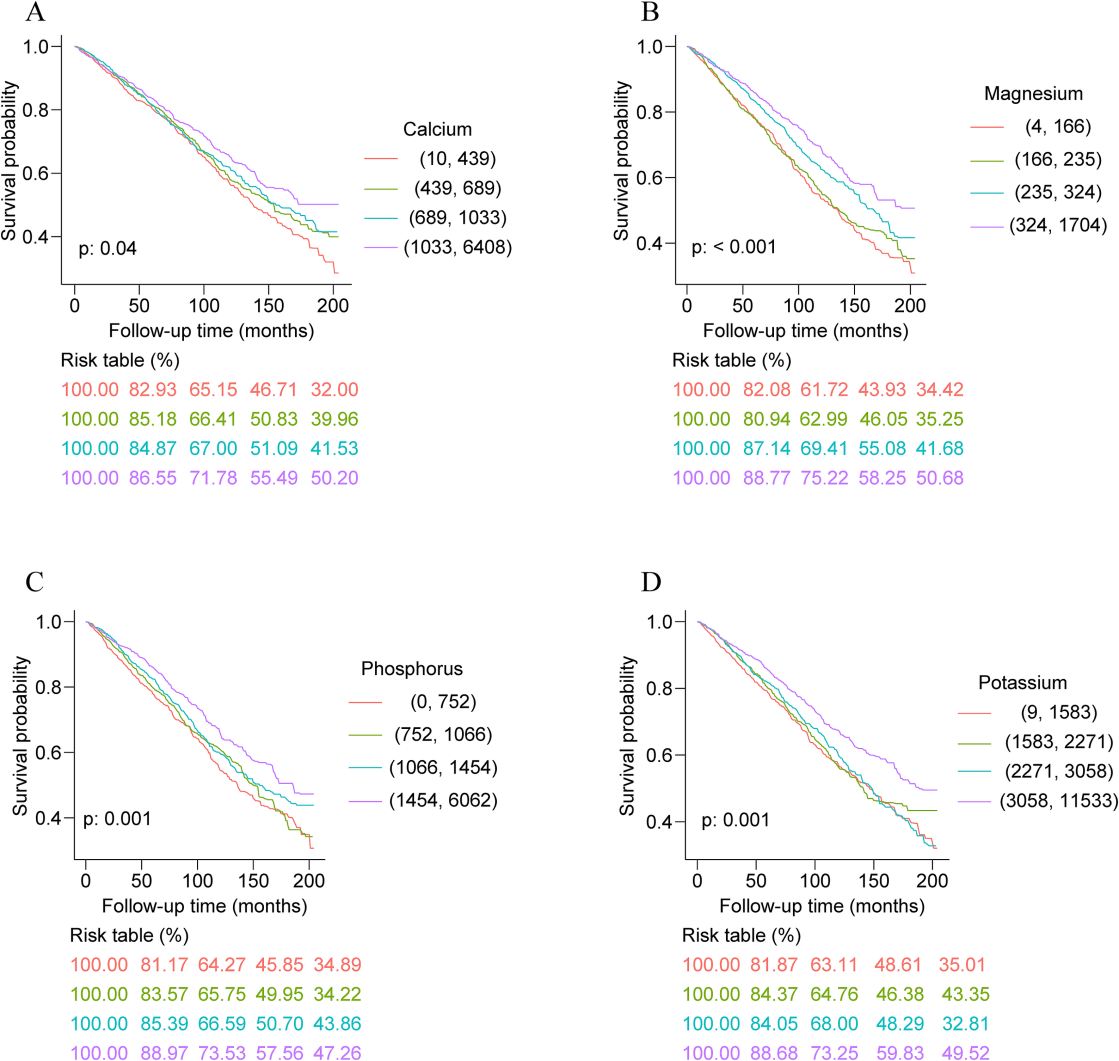
Kaplan–Meier survival analysis curves of dietary intakes of calcium **(A)**, magnesium **(B)**, phosphorus **(C)**, and potassium **(D)** and all-cause mortality.

Over 6.25 years of median follow-up, an aggregate of 1,582 deaths occurred among the study participants. Kaplan–Meier survival analyses uncovered that in individuals with ASCVD, significant statistical differences in all-cause mortality were observed across quartile groups for the intake levels of dietary calcium, magnesium, phosphorus, potassium, sodium, zinc, copper, and selenium (*p* for log-rank test <0.05; Figure 1; Supplementary Figure S2). This indicates clear associations between higher intake of these minerals and variations in all-mortality risk. However, no significant differences in all-cause mortality were detected across quartiles for dietary iron intake (*p* for log-rank test = 0.19; Supplementary Figure S3).

### 3.3 Dietary minerals intake and outcome in individuals with ASCVD

We employed the point-biserial method to preliminarily assess the correlation between the intake of nine dietary minerals and the occurrence of ASCVD (Supplementary Table S1). In the initial model (Model 1), without adjustments for covariates, direct linear relationships were identified between the intake levels of dietary zinc, calcium, potassium, magnesium, copper, phosphorus, sodium, and selenium, and all-cause mortality risk (*p* for trend <0.05) as presented in Table 2. This preliminary finding indicated potential links between the consumption of these dietary minerals and mortality outcomes.

**Table 2:** HR (95% CI) for all-cause mortality across groups of dietary minerals intake.

Characteristics	Quartiles of dietary minerals intake				*p* for trend
	Quartile 1	Quartile 2	Quartile 3	Quartile 4	
Calcium					
Range	(10, 439)	(439, 689)	(689, 1,033)	(1,033, 6,408)	
No. Deaths, n	422	396	405	359	
Model 1	1.00	0.89 (0.74, 1.07)	0.86 (0.74, 1.01)	0.75 (0.62, 0.91)	0.004
Model 2	1.00	0.88 (0.74, 1.06)	0.93 (0.78, 1.12)	0.88 (0.69, 1.12)	0.39
Model 3	1.00	0.88 (0.74, 1.06)	0.95 (0.79, 1.15)	0.87 (0.69, 1.10)	0.39
Iron					
Range	(0.02, 8.27)	(8.27, 11.92)	(11.92, 17.14)	(17.14, 111.04)	
No. Deaths, n	377	408	406	391	
Model 1	1.00	0.92 (0.75, 1.13)	0.94 (0.78, 1.13)	0.79 (0.63, 0.99)	0.05
Model 2	1.00	0.81 (0.67, 0.99)	0.82 (0.66, 1.02)	0.69 (0.54, 0.89)	0.01
Model 3	1.00	0.80 (0.66, 0.98)	0.82 (0.67, 1.02)	0.69 (0.54, 0.88)	0.01
Magnesium					
Range	(4, 166)	(166, 235)	(235, 324)	(324, 1704)	
No. Deaths, n	430	439	389	324	
Model 1	1.00	0.96 (0.83, 1.11)	0.75 (0.62, 0.91)	0.62 (0.52, 0.75)	< 0.0001
Model 2	1.00	0.89 (0.76, 1.04)	0.73 (0.60, 0.89)	0.64 (0.51, 0.82)	< 0.001
Model 3	1.00	0.88 (0.75, 1.03)	0.72 (0.58, 0.89)	0.63 (0.49, 0.81)	< 0.001
Phosphorus					
Range	(0, 752)	(752, 1,066)	(1,066, 1,454)	(1,454, 6,062)	
No. Deaths, n	420	415	408	339	
Model 1	1.00	0.90 (0.76, 1.08)	0.83 (0.68, 1.02)	0.68 (0.57, 0.82)	< 0.0001
Model 2	1.00	1.00 (0.83, 1.21)	0.89 (0.71, 1.13)	0.84 (0.63, 1.11)	0.16
Model 3	1.00	0.98 (0.82, 1.18)	0.86 (0.68, 1.09)	0.78 (0.60, 1.03)	0.06
Potassium					
Range	(9, 1,583)	(1,583, 2,271)	(2,271, 3,058)	(3,058, 11,533)	
No. Deaths, n	403	406	419	354	
Model 1	1.00	0.93 (0.76, 1.13)	0.92 (0.75, 1.12)	0.68 (0.56, 0.84)	< 0.001
Model 2	1.00	0.83 (0.70, 0.98)	0.82 (0.66, 1.01)	0.70 (0.55, 0.90)	0.01
Model 3	1.00	0.83 (0.70, 0.99)	0.78 (0.62, 0.98)	0.66 (0.51, 0.85)	0.003
Sodium					
Range	(5, 1,908)	(1,908, 2,708)	(2,708, 3,776)	(3,776, 18,053)	
No. Deaths, n	443	419	396	324	
Model 1	1.00	0.80 (0.66, 0.97)	0.68 (0.54, 0.85)	0.56 (0.45, 0.69)	< 0.0001
Model 2	1.00	0.82 (0.68, 0.99)	0.73 (0.56, 0.94)	0.71 (0.54, 0.94)	0.01
Model 3	1.00	0.79 (0.66, 0.95)	0.69 (0.54, 0.89)	0.67 (0.51, 0.88)	0.002
Zinc					
Range	(0.02, 5.8)	(5.8, 8.56)	(8.56, 12.75)	(12.75, 279.36)	
No. Deaths, n	395	417	390	380	
Model 1	1.00	0.96 (0.76, 1.21)	0.83 (0.67, 1.03)	0.74 (0.60, 0.90)	< 0.001
Model 2	1.00	0.91 (0.72, 1.13)	0.79 (0.63, 1.00)	0.80 (0.63, 1.02)	0.04
Model 3	1.00	0.89 (0.72, 1.10)	0.78 (0.62, 0.98)	0.79 (0.63, 1.00)	0.03
Copper					
Range	(0.017, 0.688)	(0.688, 0.987)	(0.987, 1.366)	(1.366, 27.364)	
No. Deaths, n	397	432	412	341	
Model 1	1.00	1.06 (0.90, 1.25)	0.74 (0.61, 0.91)	0.66 (0.54, 0.80)	< 0.0001
Model 2	1.00	0.95 (0.80, 1.12)	0.67 (0.54, 0.83)	0.64 (0.50, 0.83)	< 0.0001
Model 3	1.00	0.93 (0.79, 1.11)	0.64 (0.51, 0.81)	0.64 (0.49, 0.83)	< 0.0001
Selenium					
Range	(0, 59.2)	(59.2, 86.1)	(86.1, 122.1)	(122.1, 593.1)	
No. Deaths, n	434	423	385	340	
Model 1	1.00	0.82 (0.67, 1.00)	0.76 (0.63, 0.91)	0.61 (0.52, 0.72)	< 0.0001
Model 2	1.00	0.86 (0.71, 1.05)	0.84 (0.68, 1.03)	0.79 (0.64, 0.96)	0.02
Model 3	1.00	0.86 (0.71, 1.03)	0.82 (0.66, 1.01)	0.79 (0.64, 0.97)	0.03

HR, hazard ratio; CI, confidence interval. Data are presented as HR (95% CI) unless indicated otherwise. Model 1: crude model; Model 2: adjusted for age, sex, race/ethnicity, education, smoking status, drinking status, leisure-time physical activity, body mass index, and energy intake; Model 3: adjusted for age, sex, race/ethnicity, education, smoking status, drinking status, leisure-time physical activity, body mass index, energy intake, total cholesterol, high-density lipoprotein, antihypertensive drug, antihyperlipidemic drug, diabetes, hypertension, and hyperlipidemia.

Upon adjusting for demographic factors and lifestyle risk factors, the analysis indicated that, apart from dietary calcium and phosphorus intake, which did not exhibit statistically significant linear relationships with all-cause mortality (*p* for trend >0.05), consumption of the other seven dietary minerals demonstrated statistically significant linear correlations with all-cause mortality (*p* for trend <0.05; Table 2).

After additional adjustments for lipid profiles, drug usage, and chronic disease statuses, we refined these associations. This analysis revealed linear relationships between dietary consumption of zinc, potassium, copper, iron, sodium, magnesium, and selenium and all-cause mortality (*p* for trend <0.05). Significantly, the analysis pointed to statistically significant disparities in mortality risk within the fourth quartile of selenium intake in relation to the reference group. Moreover, notable mortality variations were present in both the second and highest quartiles of iron consumption. The analysis extended to reveal statistically significant mortality risk within the third and highest quartiles for the consumption of zinc, magnesium, and copper in relation to the reference group. In addition, a comprehensive examination unveiled statistically significant variances in mortality risk across quartiles of potassium and sodium intake when benchmarked against the reference group (Table 2).

### 3.4 Stratified and sensitivity analyses

Stratified analyses were conducted to identify potential modifiers that influence the connections between dietary minerals ingestion and mortality outcomes among individuals with ASCVD. The investigation identified BMI as a significant determinant influencing these associations, with statistical analyses demonstrating significant interactions (*p* for interaction <0.05; Supplementary Table S2). Additionally, hypertension and diabetes were pinpointed as specific factors that alter the associations between dietary intake of phosphorus and sodium, respectively, and mortality outcomes. Furthermore, the synergistic impact of age and diabetes on modulating the relationship between consuming dietary copper and mortality was identified as critical.

In sensitivity assessments that removed subjects who died within the initial two-year observation period, consistent negative linear relationships between dietary consumption of magnesium, potassium, and copper and all-cause mortality were observed, as evidenced by three distinct Cox analytical models (*p* for trend <0.05; Supplementary Table S3). Further stratification of dietary minerals intake into quintiles revealed sustained linear negative correlations with all-cause mortality for magnesium, potassium, sodium, and copper, both in models without adjustments and in those adjusted for covariates (*p* for trend <0.05; Supplementary Table S4). Additionally, the inclusion of two liver function markers, alanine aminotransferase and aspartate aminotransferase, alter the linear negative correlations between the intake of these minerals and mortality outcome (*p* for trend <0.05; Supplementary Table S5).

### 3.5 Nonlinear and linear relationships between dietary minerals intake and mortality with restricted cubic splines

**Figure 2:**
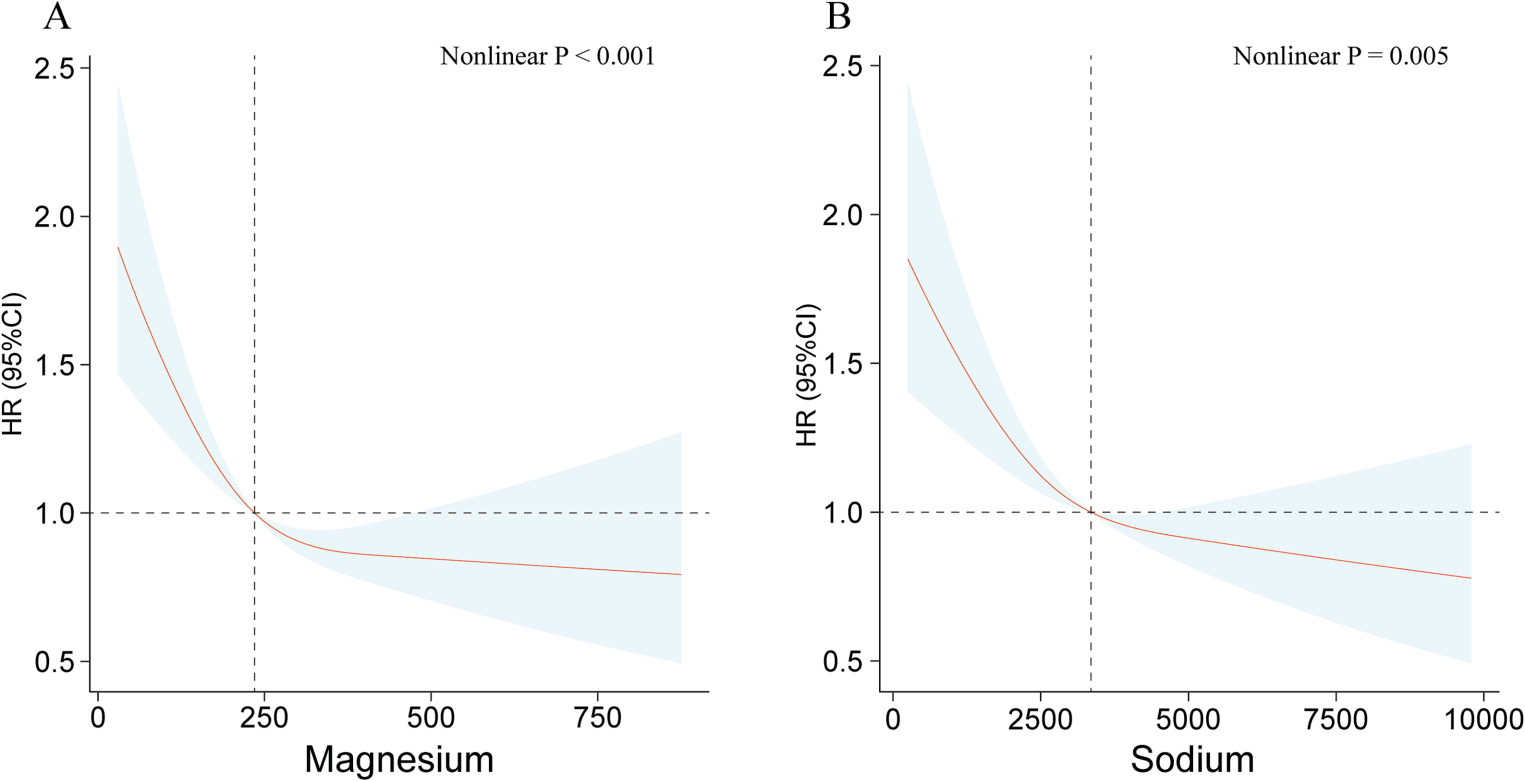
The relationships between dietary intakes of magnesium **(A)** and sodium **(B)** and all-cause mortality using the restricted cubic spline method.

Considering the potential nonlinear and linear connections between ingestion of minerals from diet and mortality outcome, as identified by weighted Cox proportional hazards regression models. We employed weighted RCS and adjusted for potential covariates to investigate these relationships further. The application of weighted RCS analyses indicated negative linear associations between dietary intake of magnesium (nonlinear *p* < 0.001) and sodium (nonlinear *p* = 0.005) and all-cause mortality (Figure 2). In contrast, dietary intake of calcium, iron, phosphorus, and selenium were observed to demonstrate J-shaped associations with all-cause mortality (Supplementary Figure S4). Furthermore, the intake of potassium, zinc, and copper showed approximate inverse L-shaped correlations with all-cause mortality (Supplementary Figure S5).

### 3.6 Stratified analyses by BMI

Results from stratified analyses distinctly highlight the pivotal role of BMI in modulating the associations between intake of various dietary minerals and all-cause mortality risk. This led to an in-depth exploration of the effects exerted by distinct BMI classifications—under/normal weight (<25 kg/m^2^), overweight (≥25 to <30 kg/m^2^), and obesity (≥30 kg/m^2^)—on this crucial health outcome.

Kaplan–Meier survival analyses provided insight that, in individuals categorized as underweight or normal weight, intake of six specific dietary minerals, excluding calcium, iron, and zinc, were significantly correlated with all-cause mortality (*p* for log-rank test <0.05). Furthermore, analyses revealed the statistically significant connections between the ingestion of nine minerals from diet and mortality within the overweight group. In addition, among the obese population, only the ingestion of sodium from diet was significantly connected with mortality outcome (Supplementary Figures S6–S8).

Utilizing Cox regression models, rigorously adjusted for all potential covariates, our study further explored the impact of BMI categories on the connections between dietary minerals consumption and the risk of mortality, with findings summarized in Supplementary Table S6. Significant relationships were identified between the intake of iron, magnesium, potassium, and copper and mortality outcomes across underweight, normal weight, and overweight individuals (*p* for trend <0.05). For individuals classified as underweight or normal weight, linear associations with mortality were also detected for the ingestion of phosphorus, zinc, and selenium (*p* for trend <0.05). Specifically, a direct linear relationship between dietary calcium ingestion and outcome was detected within the overweight population (*p* for trend = 0.041). Moreover, a consistent linear connection between dietary sodium consumption and outcome was established across the spectrum of underweight, normal weight, and obese groups, highlighting the pervasive impact of sodium intake on health outcomes (under/normal weight: *p* for trend = 0.032; obesity: *p* for trend = 0.015).

**Figure 3:**
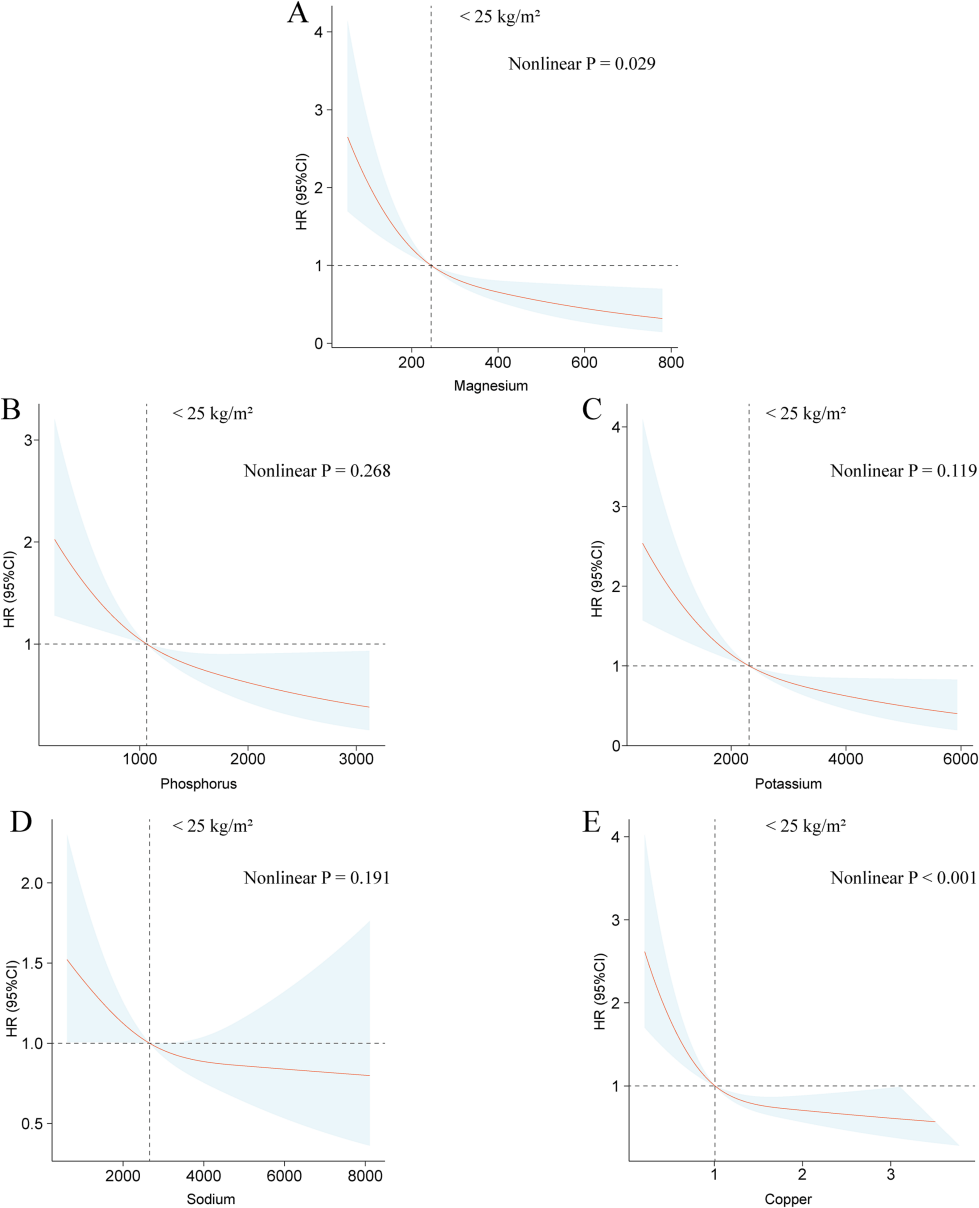
The associations between dietary intakes of magnesium **(A)**, phosphorus **(B)**, potassium **(C)**, sodium **(D)**, and copper **(E)** and all-cause mortality within populations of normal or low body weight using the restricted cubic spline method.

Analyses using restricted cubic splines showed that among individuals classified as low or normal weight, there are linear associations between the intake of dietary magnesium, phosphorus, potassium, sodium, and copper and all-cause mortality (Figure 3). For those categorized as underweight or normal weight, significant nonlinear associations were identified for dietary iron, zinc, and selenium intake with mortality outcome, with the exception of calcium (Supplementary Figure S9). In the overweight population, linear associations were observed between the intake of dietary calcium, iron, magnesium, phosphorus, potassium, and sodium and mortality from any cause (Supplementary Figure S10). Conversely, a statistically significant nonlinear relationship was found between dietary copper consumption and mortality from any cause in the overweight demographic (Supplementary Figure S11). Nonlinear associations between the consumption of dietary zinc and selenium and mortality from any cause were not statistically significant in the overweight population (Supplementary Figure S11). Among the obese population, dietary sodium consumption was linearly connected with mortality from any cause, while intake of zinc, potassium, magnesium, copper, phosphorus, and selenium exhibited statistically significant nonlinear associations (Supplementary Figure S12). The nonlinear relationships between dietary calcium and iron consumption and mortality outcome were not found to be statistically significant (Supplementary Figure S12).

## 4 Discussion

This comprehensive, representative longitudinal cohort study marks the initial investigation into the links between consumption of dietary minerals and the risk of all-cause mortality among individuals with ASCVD. Utilizing Kaplan–Meier survival curves and Cox proportional hazards models, our results indicate that elevated consumption of dietary magnesium, potassium, sodium, zinc, copper, and selenium correlates with a decreased all-cause mortality risk in individuals with ASCVD. The consistency of these associations has been further validated through various sensitivity analyses, particularly highlighting the robust relationships between magnesium, potassium, and copper intake and mortality risk. Additionally, restricted cubic spline analyses have clarified linear inverse correlations for magnesium and sodium intake related to mortality from any cause. Consequently, the convergence of evidence from multiple analytical approaches, demonstrating a consistent linear inverse relationship between magnesium consumption from diet and the risk of all-cause mortality in individuals with ASCVD, substantiates the reliability and coherence of our findings.

Stratified analyses have identified the significant role of BMI in modulating the relationships between minerals intake from diet and the risk of all-cause mortality, necessitating further examination across distinct BMI categories. Employing Kaplan–Meier survival curves, Cox proportional hazards models, and RCS analysis, this study reveals that for individuals classified as underweight or of normal weight, intake of dietary magnesium, phosphorus, potassium, sodium, and copper significantly inversely correlates with all-cause mortality risk. Conversely, within the overweight population, statistically significant inverse correlations are observed with the consumption of dietary iron, magnesium, calcium, and potassium. Notably, within the obese population, only dietary sodium intake exhibits a significant negative correlation with mortality risk.

The relationship between magnesium consumption from diet and the risk of all-cause mortality has been thoroughly examined among varied populations. A comprehensive meta-analysis, including 1,168,756 participants, demonstrated that magnesium consumption in diet is negatively linked to mortality from any cause, a correlation not observed with magnesium supplement usage (20). In an examination of the general adult demographic in the United States, utilizing data from NHANES 1999 to 2010, Chen and colleagues discovered a significant inverse association between food-based magnesium intake and all-cause mortality risk (21). Similarly, within the Japanese study, which included 58,615 middle-aged and elderly Japanese subjects, Zhang and colleagues discovered that the consumption of dietary magnesium among women was significantly linked to a reduced mortality risk in those with cardiovascular diseases (16). Further studies have highlighted that in the diabetic population, a higher consumption of dietary magnesium is connected with a lower risk of mortality from any cause (22). The Dutch Alpha Omega prospective cohort study further emphasizes the critical role of sufficient dietary magnesium consumption in lowering mortality from any cause in myocardial infarction survivors (23). Wang et al. observed that in stroke survivors, adequate consumption of dietary magnesium is inversely connected with mortality risk, with this association being statistically significant, particularly among women, those with adequate energy intake, non-hypertensives, non-smokers, and individuals with normal renal function (24). A randomized controlled trial in the United States discovered a reverse relationship between magnesium consumption from diet and the risk of all-cause mortality in individuals with a high risk of cardiovascular conditions (25). Results from a randomized controlled trial suggested that magnesium intake significantly enhances serum 25-hydroxyvitamin D levels in overweight and obese individuals (26). Additionally, a notable association was identified between serum 25-hydroxyvitamin D concentrations and all-cause mortality (27). Conversely, a study in Taiwan did not uncover a significant connection between magnesium intake and diet, and it reduced all-cause mortality risk among the elderly, possibly due to distinct dietary patterns and the small sample size (28). In this extensive prospective cohort study, after adjusting for multiple covariates in 4,125 individuals with ASCVD, we identified a statistically significant linear inverse connection between magnesium intake from diet and mortality outcome, aligning with the findings from the aforementioned research.

Several potential mechanisms have been proposed to clarify the decreased risk of all-cause mortality in individuals with ASCVD attributed to dietary magnesium consumption. Magnesium plays a crucial function in adjusting diverse factors implicated in the development of atherosclerosis, including lipid metabolism, oxidative stress, inflammatory response, and the function of endothelial cells. Research indicates that magnesium ions can bind to lipoproteins, facilitating an interactive relationship that influences lipid profiles (29). Magnesium supplementation has been shown to significantly ameliorate dyslipidemia, a key contributor to atherosclerosis, by reducing total cholesterol, triglycerides, low-density lipoproteins, and very low-density lipoproteins (30–32). Furthermore, magnesium’s capacity to regulate intracellular calcium overload, reduce cellular oxygen demand, and mitigate oxidative stress highlights its crucial role in vascular health (33). Magnesium ions, predominantly located in mitochondria, are vital for cellular function. A deficiency in magnesium results in increased generation of reactive oxygen species and mitochondrial dysfunction, impairing the antioxidant defense mechanism and inducing oxidative stress in vascular endothelial cells. This oxidative stress promotes endothelial dysfunction, facilitating intracellular lipid accumulation and atherosclerosis progression (34, 35). Magnesium deficiency exacerbates oxidative stress, contributing to endothelial cell impairment and the initiation of atherosclerotic processes (36). Additionally, increased concentrations of interleukin-1 and interleukin-6, key pro-inflammatory cytokines, correlate with endothelial impairment and the development of atherosclerosis (37). Magnesium acts as a potent anti-inflammatory agent, exerting anti-atherosclerotic effects by suppressing the release of pro-inflammatory factors (37).

Subgroup analysis, stratified according to BMI levels, has uncovered significant correlations between consumption of dietary minerals and risk of mortality from any cause, particularly pronounced in underweight, normal weight, and overweight populations. Existing literature indicates that obese individuals often do not meet the recommended dietary minerals levels (38). Specifically, studies have shown that dietary calcium intake is lower in overweight than in normal-weight individuals, magnesium consumption is reduced in obese populations, and potassium intake is inversely related to BMI levels (39–42). Our study has identified linear negative relationships between the intake of dietary magnesium and potassium with all-cause mortality risk in overweight populations and those with a lower BMI, suggesting that these associations may be influenced by the amount of dietary minerals consumption. A comprehensive meta-analysis, incorporating prospective studies from four continents, established a connection between BMI and the risk of mortality from any cause, particularly among overweight and obese individuals (43). Additionally, a Mendelian randomization analysis employing genome-wide association data confirmed a direct link between BMI and the risk of mortality from any cause (44). In a large-scale study by Wang et al., involving 1,300,794 participants, a J-shaped association was detected between BMI and the risk of mortality in individuals with coronary artery disease (45), indicating that different BMI categories distinctly influence the connections between minerals intake from diet and mortality risk.

The strength of this research lies in its utilization of a large, representative cohort and the application of multiple analytical techniques, which elucidate the impacts of nine dietary minerals on all-cause mortality risk, providing a comprehensive and in-depth analysis. Moreover, data were collected by professionally trained staff following a rigorous research protocol and quality control processes, ensuring the precision of the findings. The examination of the relationships between dietary minerals intake and all-cause mortality risk across different BMI levels offers personalized insights for the application of our study findings.

Our study, while providing valuable insights into the prognostic implications of dietary minerals intake, comes with certain limitations that merit acknowledgment. Initially, our investigation centers on the baseline intake of nine distinct dietary minerals, leaving the impact of fluctuations in dietary minerals intake over the course of the follow-up period an area ripe for further exploration. This gap highlights the need for longitudinal studies to assess how changes in dietary habits may affect mortality risk over time. Additionally, the dietary patterns of our study cohort are reflective of those prevalent within the American context, predominantly influenced by Western eating habits. This specificity in dietary culture may restrict the applicability of our findings to populations with analogous dietary behaviors, thus potentially limiting their relevance to communities with distinct dietary traditions, such as those found in Eastern cultures. Moreover, despite our diligent efforts to control for a range of confounding variables through multivariate adjustments and stratified analysis, the observational design of our study inherently carries the risk of residual confounding. This limitation suggests that there could be other influential factors not identified or measured in our study that may affect the connections between minerals intake from diet and mortality risk. Finally, the definition of ASCVD in NHANES relies on self-reported data from standardized questionnaires, which might introduce some degree of reporting bias. As such, these considerations should guide the interpretation of our results, advocating for a cautious approach in extrapolating these findings beyond the studied population.

## 5 Conclusion

In this representative prospective cohort study, it was found that solely magnesium intake, in contrast to other evaluated dietary minerals, is linearly inversely connected with all-cause mortality risk in individuals with ASCVD. Furthermore, BMI serves as a critical factor potentially affecting the relationship between the intake of dietary minerals such as iron, phosphorus, calcium, sodium, magnesium, potassium, and copper and the risk of all-cause mortality.

## Data Availability

The original contributions presented in the study are included in the article/Supplementary material, further inquiries can be directed to the corresponding author.
