# Supplementary material for: Associations between nine dietary minerals intake and all-cause mortality in individuals with atherosclerotic cardiovascular disease
Source: Front Nutr. 2024 Oct 14;11:1447167. doi: 10.3389/fnut.2024.1447167 (PMC11513590; doi:10.3389/fnut.2024.1447167)
Supplement: Supplementary file 1 [file Data_Sheet_1.docx]

**Supplementary materials**

[**Supplementary Tables** 2](#_Toc178060811)

[**Supplementary Table S1.** The correlation between nine dietary minerals and ASCVD using Point-Biserial method. 2](#_Toc178060812)

[**Supplementary Table S2**. Stratified analyses of the associations between dietary minerals intake and all-cause mortality among individuals with ASCVD 4](#_Toc178060813)

[**Supplementary Table S3**. HRs (95% CIs) of all-cause mortality according to dietary minerals intake after excluding ASCVD individuals with less than 2 years of follow-up. 11](#_Toc178060814)

[**Supplementary Table S4**. HR (95% CIs) of all-cause mortality according to quintiles of dietary mineral intake among individuals with ASCVD. 14](#_Toc178060815)

[**Supplementary Table S5**. HRs (95% CIs) of all-cause mortality according to dietary minerals intake among ASCVD individuals with further adjustment of several liver function biomarkers. 17](#_Toc178060816)

[**Supplementary Table S6**. Subgroup analyses for all-cause mortality according to dietary minerals intake after stratification by BMI 20](#_Toc178060817)

[**Supplementary Figures** 23](#_Toc178060818)

[**Supplementary Figure S1**. The flow chart of participants inclusion and exclusion in this study. 23](#_Toc178060819)

[**Supplementary Figure S2**. Kaplan-Meier survival analysis curves of dietary intakes of sodium (A), zinc (B), copper (C), and selenium (D) and all-cause mortality. 24](#_Toc178060820)

[**Supplementary Figure S3**. Kaplan-Meier survival analysis curve of dietary intake of iron and all-cause mortality. 25](#_Toc178060821)

[**Supplementary Figure S4**. The relationships between dietary intakes of calcium (A), iron (B), phosphorus (C), and selenium (D), and all-cause mortality using restricted cubic spline method. 26](#_Toc178060822)

[**Supplementary Figure S5**. The relationships between dietary intakes of potassium (A), zinc (B), and copper (C), and all-cause mortality using restricted cubic spline method. 27](#_Toc178060823)

[**Supplementary Figure S6.** Kaplan-Meier survival analysis curves of dietary intakes of calcium (A, B, C), iron (D, E, F), and magnesium (G, H, I), and all-cause mortality after stratification by BMI. 28](#_Toc178060824)

[**Supplementary Figure S7.** Kaplan-Meier survival analysis curves of dietary intakes of phosphorus (A, B, C), potassium (D, E, F), and sodium (G, H, I), and all-cause mortality after stratification by BMI. 29](#_Toc178060825)

[**Supplementary Figure S8.** Kaplan-Meier survival analysis curves of dietary intakes of zinc (A, B, C), copper (D, E, F), and selenium (G, H, I), and all-cause mortality after stratification by BMI. 30](#_Toc178060826)

[**Supplementary Figure S9**. The associations between dietary intakes of calcium(A), iron (B), zinc (C), and selenium (D) and all-cause mortality within populations of normal or low body weight using restricted cubic spline method. 31](#_Toc178060827)

[**Supplementary Figure S10.** The associations between dietary intakes of calcium(A), iron (B), magnesium (C), phosphorus (D), potassium (E), and sodium (F) and all-cause mortality within populations of overweight using restricted cubic spline method. 32](#_Toc178060828)

[**Supplementary Figure S11.** The associations between dietary intakes of zinc(A), copper (B), and selenium (C) and all-cause mortality within populations of overweight using restricted cubic spline method. 33](#_Toc178060829)

[**Supplementary Figure S12.** The associations between dietary intakes of calcium(A), iron (B), magnesium (C), phosphorus (D), potassium (E), sodium (F), zinc (G), copper (H), and selenium (I) and all-cause mortality within populations of obesity using restricted cubic spline method. 34](#_Toc178060830)

[**Supplementary method** 35](#_Toc178060831)

# **Supplementary Tables**

## **Supplementary Table S1.** The correlation between nine dietary minerals and ASCVD using Point-Biserial method.

| Characteristics | Correlation coefficient | P-value |
| --- | --- | --- |
| Calcium | -0.104 | < 0.001 |
| Iron | -0.068 | < 0.001 |
| Magnesium | -0.128 | < 0.001 |
| Phosphorus | -0.144 | < 0.001 |
| Potassium | -0.087 | < 0.001 |
| Sodium | -0.131 | < 0.001 |
| Zinc | -0.072 | < 0.001 |
| Copper | -0.070 | < 0.001 |
| Selenium | -0.132 | < 0.001 |

ASCVD, atherosclerotic cardiovascular diseases.

## **Supplementary Table S2**. Stratified analyses of the associations between dietary minerals intake and all-cause mortality among individuals with ASCVD

| Characteristics | Quartiles of dietary minerals intake | | | | *P* for trend | *P* for interaction |
| --- | --- | --- | --- | --- | --- | --- |
|  | Quartile 1 | Quartile 2 | Quartile 3 | Quartile 4 |  |  |
| **Calcium** | | | | | | |
| Range | (10, 439) | (439, 689) | (689, 1,033) | (1,033, 6,408) |  |  |
| Age |  |  |  |  |  | 0.241 |
| < 60 | 1.000 | 0.673 (0.374, 1.211) | 0.563 (0.322, 0.984) | 0.675 (0.344, 1.326) | 0.229 |  |
| ≥ 60 | 1.000 | 0.950 (0.793, 1.137) | 1.052 (0.867, 1.277) | 0.930 (0.718, 1.205) | 0.816 |  |
| Sex |  |  |  |  |  | 0.652 |
| Male | 1.000 | 0.860 (0.643, 1.151) | 0.841 (0.634, 1.117) | 0.794 (0.563, 1.120) | 0.2 |  |
| Female | 1.000 | 0.926 (0.724, 1.183) | 1.156 (0.896, 1.492) | 1.053 (0.723, 1.535) | 0.521 |  |
| BMI |  |  |  |  |  | 0.003 |
| < 30 | 1.000 | 0.905 (0.711, 1.154) | 0.908 (0.718, 1.148) | 0.788 (0.605, 1.027) | 0.109 |  |
| ≥ 30 | 1.000 | 0.873 (0.650, 1.172) | 1.012 (0.716, 1.430) | 1.022 (0.675, 1.550) | 0.722 |  |
| Hypertension |  |  |  |  |  | 0.113 |
| No | 1.000 | 0.710 (0.400, 1.261) | 1.165 (0.791, 1.718) | 0.843 (0.508, 1.401) | 0.949 |  |
| Yes | 1.000 | 0.924 (0.778, 1.099) | 0.914 (0.750, 1.114) | 0.899 (0.709, 1.140) | 0.389 |  |
| Diabetes |  |  |  |  |  | 0.786 |
| No | 1.000 | 0.851 (0.657, 1.103) | 1.016 (0.795, 1.298) | 0.933 (0.698, 1.247) | 0.952 |  |
| Yes | 1.000 | 0.904 (0.731, 1.118) | 0.906 (0.693, 1.186) | 0.800 (0.571, 1.121) | 0.218 |  |
| **Iron** | | | | | | |
| Range | (0.02, 8.27) | (8.27, 11.92) | (11.92, 17.14) | (17.14, 111.04) |  |  |
| Age |  |  |  |  |  | 0.176 |
| < 60 | 1.000 | 0.671 (0.383, 1.176) | 0.940 (0.469, 1.882) | 0.566 (0.217, 1.481) | 0.339 |  |
| ≥ 60 | 1.000 | 0.911 (0.748, 1.110) | 0.951 (0.781, 1.157) | 0.826 (0.646, 1.057) | 0.185 |  |
| Sex |  |  |  |  |  | 0.764 |
| Male | 1.000 | 0.870 (0.651, 1.162) | 0.831 (0.621, 1.113) | 0.685 (0.470, 0.999) | 0.046 |  |
| Female | 1.000 | 0.795 (0.594, 1.064) | 0.894 (0.659, 1.211) | 0.754 (0.549, 1.036) | 0.155 |  |
| BMI |  |  |  |  |  | < 0.001 |
| < 30 | 1.000 | 0.735 (0.572, 0.944) | 0.780 (0.598, 1.017) | 0.581 (0.435, 0.778) | 0.001 |  |
| ≥ 30 | 1.000 | 0.931 (0.661, 1.313) | 0.872 (0.552, 1.377) | 0.910 (0.581, 1.426) | 0.67 |  |
| Hypertension |  |  |  |  |  | 0.751 |
| No | 1.000 | 0.787 (0.515, 1.203) | 0.901 (0.577, 1.406) | 0.706 (0.378, 1.317) | 0.384 |  |
| Yes | 1.000 | 0.819 (0.665, 1.008) | 0.817 (0.648, 1.031) | 0.697 (0.541, 0.898) | 0.011 |  |
| Diabetes |  |  |  |  |  | 0.49 |
| No | 1.000 | 0.736 (0.553, 0.980) | 0.788 (0.594, 1.047) | 0.631 (0.464, 0.858) | 0.013 |  |
| Yes | 1.000 | 0.898 (0.664, 1.216) | 0.856 (0.616, 1.189) | 0.745 (0.535, 1.036) | 0.077 |  |
| **Magnesium** | | | | | | |
| Range | (4, 166) | (166, 235) | (235, 324) | (324, 1,704) |  |  |
| Age |  |  |  |  |  | 0.162 |
| < 60 | 1.000 | 1.125 (0.657, 1.925) | 0.630 (0.331, 1.200) | 0.660 (0.303, 1.439) | 0.194 |  |
| ≥ 60 | 1.000 | 0.921 (0.771, 1.100) | 0.794 (0.643, 0.981) | 0.681 (0.508, 0.913) | 0.005 |  |
| Sex |  |  |  |  |  | 0.478 |
| Male | 1.000 | 0.989 (0.784, 1.247) | 0.740 (0.545, 1.006) | 0.642 (0.455, 0.905) | 0.002 |  |
| Female | 1.000 | 0.838 (0.658, 1.067) | 0.801 (0.561, 1.143) | 0.695 (0.440, 1.097) | 0.118 |  |
| BMI |  |  |  |  |  | < 0.0001 |
| < 30 | 1.000 | 0.798 (0.665, 0.958) | 0.620 (0.484, 0.794) | 0.485 (0.361, 0.650) | < 0.0001 |  |
| ≥ 30 | 1.000 | 1.011 (0.713, 1.432) | 0.905 (0.596, 1.374) | 0.963 (0.580, 1.599) | 0.773 |  |
| Hypertension |  |  |  |  |  | 0.087 |
| No | 1.000 | 0.695 (0.478, 1.009) | 0.724 (0.436, 1.202) | 0.472 (0.263, 0.850) | 0.026 |  |
| Yes | 1.000 | 0.906 (0.745, 1.102) | 0.717 (0.570, 0.902) | 0.680 (0.518, 0.893) | 0.002 |  |
| Diabetes |  |  |  |  |  | 0.681 |
| No | 1.000 | 0.920 (0.740, 1.144) | 0.791 (0.626, 0.999) | 0.663 (0.478, 0.919) | 0.008 |  |
| Yes | 1.000 | 0.861 (0.673, 1.102) | 0.691 (0.497, 0.960) | 0.620 (0.415, 0.927) | 0.009 |  |
| **Phosphorus** | | | | | | |
| Range | (0, 752) | (752, 1,066) | (1,066, 1,454) | (1,454, 6,062) |  |  |
| Age |  |  |  |  |  | 0.615 |
| < 60 | 1.000 | 0.965 (0.499, 1.865) | 0.841 (0.387, 1.829) | 0.718 (0.308, 1.672) | 0.407 |  |
| ≥ 60 | 1.000 | 1.035 (0.844, 1.268) | 0.921 (0.723, 1.174) | 0.853 (0.650, 1.120) | 0.205 |  |
| Sex |  |  |  |  |  | 0.508 |
| Male | 1.000 | 0.974 (0.756, 1.255) | 0.921 (0.670, 1.267) | 0.778 (0.544, 1.114) | 0.16 |  |
| Female | 1.000 | 1.123 (0.863, 1.461) | 0.928 (0.656, 1.312) | 0.956 (0.592, 1.542) | 0.66 |  |
| BMI |  |  |  |  |  | < 0.0001 |
| < 30 | 1.000 | 0.871 (0.687, 1.105) | 0.832 (0.638, 1.084) | 0.624 (0.450, 0.864) | 0.011 |  |
| ≥ 30 | 1.000 | 1.155 (0.807, 1.651) | 0.835 (0.519, 1.342) | 1.062 (0.589, 1.915) | 0.766 |  |
| Hypertension |  |  |  |  |  | 0.028 |
| No | 1.000 | 0.679 (0.417, 1.106) | 0.788 (0.495, 1.256) | 0.555 (0.311, 0.991) | 0.096 |  |
| Yes | 1.000 | 1.058 (0.847, 1.320) | 0.867 (0.669, 1.123) | 0.870 (0.626, 1.208) | 0.213 |  |
| Diabetes |  |  |  |  |  | 0.424 |
| No | 1.000 | 0.963 (0.744, 1.245) | 0.951 (0.699, 1.292) | 0.864 (0.602, 1.242) | 0.488 |  |
| Yes | 1.000 | 0.999 (0.743, 1.342) | 0.745 (0.528, 1.050) | 0.704 (0.461, 1.075) | 0.034 |  |
| **Potassium** | | | | | | |
| Range | (9, 1,583) | (1,583, 2,271) | (2,271, 3,058) | (3,058, 11,533) |  |  |
| Age |  |  |  |  |  | 0.073 |
| < 60 | 1.000 | 0.721 (0.424, 1.228) | 0.953 (0.535, 1.696) | 0.576 (0.304, 1.090) | 0.209 |  |
| ≥ 60 | 1.000 | 0.920 (0.758, 1.115) | 0.844 (0.643, 1.108) | 0.734 (0.551, 0.977) | 0.044 |  |
| Sex |  |  |  |  |  | 0.419 |
| Male | 1.000 | 0.682 (0.529, 0.881) | 0.680 (0.520, 0.889) | 0.567 (0.428, 0.750) | 0.003 |  |
| Female | 1.000 | 0.956 (0.743, 1.230) | 0.895 (0.621, 1.289) | 0.804 (0.482, 1.341) | 0.417 |  |
| BMI |  |  |  |  |  | < 0.0001 |
| < 30 | 1.000 | 0.693 (0.558, 0.860) | 0.640 (0.490, 0.836) | 0.490 (0.359, 0.669) | < 0.0001 |  |
| ≥ 30 | 1.000 | 1.064 (0.777, 1.457) | 0.992 (0.676, 1.457) | 1.029 (0.637, 1.660) | 0.997 |  |
| Hypertension |  |  |  |  |  | 0.217 |
| No | 1.000 | 0.679 (0.449, 1.025) | 0.722 (0.437, 1.190) | 0.526 (0.267, 1.036) | 0.111 |  |
| Yes | 1.000 | 0.852 (0.701, 1.036) | 0.781 (0.605, 1.009) | 0.698 (0.530, 0.920) | 0.015 |  |
| Diabetes |  |  |  |  |  | 0.655 |
| No | 1.000 | 0.810 (0.637, 1.029) | 0.791 (0.585, 1.070) | 0.658 (0.484, 0.894) | 0.016 |  |
| Yes | 1.000 | 0.857 (0.645, 1.140) | 0.813 (0.589, 1.123) | 0.720 (0.488, 1.063) | 0.119 |  |
| **Sodium** | | | | | | |
| Range | (5, 1,908) | (1,908, 2,708) | (2,708, 3,776) | (3,776, 18,053) |  |  |
| Age |  |  |  |  |  | 0.454 |
| < 60 | 1.000 | 0.671 (0.377, 1.194) | 0.739 (0.348, 1.571) | 0.675 (0.295, 1.544) | 0.499 |  |
| ≥ 60 | 1.000 | 0.742 (0.611, 0.900) | 0.708 (0.574, 0.873) | 0.634 (0.479, 0.839) | 0.001 |  |
| Sex |  |  |  |  |  | 0.549 |
| Male | 1.000 | 0.840 (0.638, 1.104) | 0.769 (0.534, 1.106) | 0.730 (0.500, 1.065) | 0.115 |  |
| Female | 1.000 | 0.807 (0.614, 1.061) | 0.712 (0.516, 0.983) | 0.670 (0.437, 1.028) | 0.026 |  |
| BMI |  |  |  |  |  | 0.002 |
| < 30 | 1.000 | 0.777 (0.626, 0.966) | 0.779 (0.573, 1.059) | 0.667 (0.482, 0.922) | 0.032 |  |
| ≥ 30 | 1.000 | 0.846 (0.626, 1.144) | 0.547 (0.372, 0.803) | 0.639 (0.405, 1.009) | 0.015 |  |
| Hypertension |  |  |  |  |  | 0.053 |
| No | 1.000 | 0.946 (0.630, 1.420) | 0.685 (0.434, 1.081) | 0.566 (0.342, 0.938) | 0.012 |  |
| Yes | 1.000 | 0.769 (0.626, 0.944) | 0.715 (0.544, 0.940) | 0.720 (0.530, 0.978) | 0.024 |  |
| Diabetes |  |  |  |  |  | 0.029 |
| No | 1.000 | 0.804 (0.621, 1.041) | 0.794 (0.576, 1.093) | 0.628 (0.443, 0.891) | 0.021 |  |
| Yes | 1.000 | 0.811 (0.602, 1.093) | 0.634 (0.436, 0.922) | 0.755 (0.494, 1.153) | 0.068 |  |
| **Zinc** | | | | | | |
| Range | (0.02, 5.8) | (5.8, 8.56) | (8.56, 12.75) | (12.75, 279.36) |  |  |
| Age |  |  |  |  |  | 0.131 |
| < 60 | 1.000 | 0.559 (0.280, 1.118) | 0.763 (0.364, 1.598) | 0.693 (0.295, 1.626) | 0.609 |  |
| ≥ 60 | 1.000 | 0.956 (0.766, 1.194) | 0.843 (0.674, 1.055) | 0.850 (0.669, 1.079) | 0.11 |  |
| Sex |  |  |  |  |  | 0.07 |
| Male | 1.000 | 0.776 (0.579, 1.040) | 0.818 (0.596, 1.121) | 0.709 (0.496, 1.014) | 0.127 |  |
| Female | 1.000 | 1.070 (0.813, 1.408) | 0.775 (0.525, 1.145) | 1.028 (0.721, 1.465) | 0.498 |  |
| BMI |  |  |  |  |  | 0.021 |
| < 30 | 1.000 | 0.974 (0.752, 1.260) | 0.775 (0.587, 1.024) | 0.831 (0.620, 1.112) | 0.091 |  |
| ≥ 30 | 1.000 | 0.787 (0.534, 1.159) | 0.788 (0.542, 1.147) | 0.736 (0.499, 1.085) | 0.184 |  |
| Hypertension |  |  |  |  |  | 0.412 |
| No | 1.000 | 1.242 (0.741, 2.084) | 0.852 (0.538, 1.352) | 1.181 (0.653, 2.135) | 0.928 |  |
| Yes | 1.000 | 0.835 (0.657, 1.059) | 0.766 (0.602, 0.974) | 0.762 (0.596, 0.974) | 0.025 |  |
| Diabetes |  |  |  |  |  | 0.666 |
| No | 1.000 | 0.864 (0.667, 1.120) | 0.739 (0.539, 1.015) | 0.753 (0.547, 1.036) | 0.057 |  |
| Yes | 1.000 | 0.927 (0.666, 1.291) | 0.819 (0.593, 1.133) | 0.843 (0.606, 1.171) | 0.205 |  |
| **Copper** | | | | | | |
| Range | (0.017, 0.688) | (0.688, 0.987) | (0.987, 1.366) | (1.366, 27.364) |  |  |
| Age |  |  |  |  |  | < 0.001 |
| < 60 | 1.000 | 1.270 (0.736, 2.191) | 0.377 (0.181, 0.787) | 0.790 (0.395, 1.579) | 0.177 |  |
| ≥ 60 | 1.000 | 0.994 (0.838, 1.180) | 0.770 (0.623, 0.952) | 0.642 (0.501, 0.822) | < 0.0001 |  |
| Sex |  |  |  |  |  | 0.371 |
| Male | 1.000 | 0.944 (0.724, 1.230) | 0.722 (0.525, 0.993) | 0.670 (0.481, 0.931) | 0.008 |  |
| Female | 1.000 | 0.971 (0.756, 1.247) | 0.597 (0.437, 0.815) | 0.640 (0.409, 0.999) | 0.005 |  |
| BMI |  |  |  |  |  | < 0.001 |
| < 30 | 1.000 | 0.971 (0.806, 1.169) | 0.610 (0.481, 0.774) | 0.555 (0.420, 0.732) | < 0.0001 |  |
| ≥ 30 | 1.000 | 0.911 (0.651, 1.277) | 0.672 (0.407, 1.112) | 0.772 (0.474, 1.258) | 0.207 |  |
| Hypertension |  |  |  |  |  | 0.135 |
| No | 1.000 | 1.254 (0.818, 1.920) | 0.652 (0.409, 1.039) | 0.661 (0.344, 1.270) | 0.06 |  |
| Yes | 1.000 | 0.881 (0.730, 1.063) | 0.640 (0.491, 0.835) | 0.630 (0.472, 0.841) | < 0.001 |  |
| Diabetes |  |  |  |  |  | 0.003 |
| No | 1.000 | 0.958 (0.760, 1.208) | 0.719 (0.554, 0.933) | 0.582 (0.429, 0.790) | < 0.001 |  |
| Yes | 1.000 | 0.973 (0.746, 1.270) | 0.570 (0.393, 0.826) | 0.738 (0.500, 1.090) | 0.014 |  |
| **Selenium** | | | | | | |
| Range | (0, 59.2) | (59.2, 86.1) | (86.1, 122.1) | (122.1, 593.1) |  |  |
| Age |  |  |  |  |  | 0.061 |
| < 60 | 1.000 | 0.594 (0.297, 1.191) | 0.850 (0.467, 1.547) | 0.520 (0.243, 1.116) | 0.246 |  |
| ≥ 60 | 1.000 | 0.838 (0.690, 1.017) | 0.806 (0.660, 0.983) | 0.772 (0.613, 0.971) | 0.017 |  |
| Sex |  |  |  |  |  | 0.208 |
| Male | 1.000 | 0.834 (0.638, 1.089) | 0.862 (0.650, 1.144) | 0.706 (0.520, 0.958) | 0.052 |  |
| Female | 1.000 | 0.931 (0.732, 1.183) | 0.850 (0.609, 1.185) | 1.098 (0.782, 1.543) | 0.879 |  |
| BMI |  |  |  |  |  | 0.045 |
| < 30 | 1.000 | 0.840 (0.645, 1.095) | 0.792 (0.598, 1.049) | 0.777 (0.589, 1.025) | 0.067 |  |
| ≥ 30 | 1.000 | 0.888 (0.638, 1.237) | 0.802 (0.524, 1.226) | 0.778 (0.487, 1.243) | 0.278 |  |
| Hypertension |  |  |  |  |  | 0.154 |
| No | 1.000 | 0.682 (0.441, 1.056) | 0.654 (0.415, 1.031) | 0.639 (0.354, 1.153) | 0.113 |  |
| Yes | 1.000 | 0.892 (0.737, 1.081) | 0.864 (0.675, 1.106) | 0.838 (0.639, 1.099) | 0.2 |  |
| Diabetes |  |  |  |  |  | 0.923 |
| No | 1.000 | 0.900 (0.691, 1.172) | 0.863 (0.653, 1.142) | 0.855 (0.664, 1.101) | 0.211 |  |
| Yes | 1.000 | 0.791 (0.598, 1.048) | 0.759 (0.550, 1.045) | 0.679 (0.465, 0.992) | 0.055 |  |

Abbreviations: HR, hazard ratio; CI, confidence interval; ASCVD, atherosclerotic cardiovascular diseases; BMI, body mass index.

Data are presented as HR (95% CI) unless indicated otherwise. Adjusted for age, sex, race/ethnicity, education, smoking status, drinking status, leisure-time physical activity, body mass index, energy intake, total cholesterol, high density lipoprotein, antihypertensive drug, antihyperlipidemic drug, diabetes, hypertension, and hyperlipidemia. The stratified variable was not included in the model when stratifying by itself.

## **Supplementary Table S3**. HRs (95% CIs) of all-cause mortality according to dietary minerals intake after excluding ASCVD individuals with less than 2 years of follow-up.

| Characteristics | Quartiles of dietary minerals intake | | | | *P* for trend |
| --- | --- | --- | --- | --- | --- |
|  | Quartile 1 | Quartile 2 | Quartile 3 | Quartile 4 |  |
| **Calcium** | | | | | |
| Range | (10, 439) | (439, 689) | (689, 1,033) | (1,033, 6,408) |  |
| Model 1 | 1.00 | 0.90 (0.74, 1.10) | 0.90 (0.75, 1.07) | 0.72 (0.58, 0.89) | 0.003 |
| Model 2 | 1.00 | 0.87 (0.72, 1.05) | 0.95 (0.77, 1.16) | 0.82 (0.63, 1.06) | 0.23 |
| Model 3 | 1.00 | 0.88 (0.72, 1.06) | 0.96 (0.78, 1.19) | 0.82 (0.63, 1.06) | 0.23 |
| **Iron** | | | | | |
| Range | (0.02, 8.27) | (8.27, 11.92) | (11.92, 17.14) | (17.14, 111.04) |  |
| Model 1 | 1.00 | 0.97 (0.76, 1.24) | 0.99 (0.80, 1.22) | 0.83 (0.66, 1.05) | 0.12 |
| Model 2 | 1.00 | 0.86 (0.68, 1.09) | 0.86 (0.69, 1.09) | 0.74 (0.58, 0.96) | 0.04 |
| Model 3 | 1.00 | 0.84 (0.66, 1.08) | 0.85 (0.68, 1.07) | 0.73 (0.56, 0.94) | 0.03 |
| **Magnesium** | | | | | |
| Range | (4, 166) | (166, 235) | (235, 324) | (324, 1,704) |  |
| Model 1 | 1.00 | 0.97 (0.81, 1.16) | 0.79 (0.64, 0.98) | 0.61 (0.50, 0.74) | < 0.0001 |
| Model 2 | 1.00 | 0.88 (0.73, 1.06) | 0.75 (0.61, 0.92) | 0.61 (0.47, 0.78) | < 0.0001 |
| Model 3 | 1.00 | 0.87 (0.71, 1.06) | 0.73 (0.58, 0.92) | 0.59 (0.45, 0.77) | < 0.0001 |
| **Phosphorus** | | | | | |
| Range | (0, 752) | (752, 1,066) | (1,066, 1,454) | (1,454, 6,062) |  |
| Model 1 | 1.00 | 0.94 (0.78, 1.14) | 0.92 (0.73, 1.16) | 0.70 (0.58, 0.84) | < 0.001 |
| Model 2 | 1.00 | 1.05 (0.87, 1.27) | 0.99 (0.77, 1.27) | 0.88 (0.66, 1.18) | 0.41 |
| Model 3 | 1.00 | 1.03 (0.85, 1.24) | 0.93 (0.73, 1.20) | 0.83 (0.62, 1.10) | 0.18 |
| **Potassium** | | | | | |
| Range | (9, 1,583) | (1,583, 2,271) | (2,271, 3,058) | (3,058, 11,533) |  |
| Model 1 | 1.00 | 1.00 (0.80, 1.24) | 0.97 (0.78, 1.20) | 0.70 (0.56, 0.88) | 0.001 |
| Model 2 | 1.00 | 0.88 (0.74, 1.05) | 0.85 (0.68, 1.06) | 0.72 (0.56, 0.93) | 0.02 |
| Model 3 | 1.00 | 0.88 (0.73, 1.05) | 0.80 (0.62, 1.02) | 0.67 (0.52, 0.88) | 0.005 |
| **Sodium** | | | | | |
| Range | (5, 1,908) | (1,908, 2,708) | (2,708, 3,776) | (3,776, 18,053) |  |
| Model 1 | 1.00 | 0.91 (0.73, 1.13) | 0.75 (0.59, 0.96) | 0.59 (0.47, 0.74) | < 0.0001 |
| Model 2 | 1.00 | 0.96 (0.77, 1.19) | 0.82 (0.62, 1.08) | 0.79 (0.58, 1.07) | 0.07 |
| Model 3 | 1.00 | 0.92 (0.75, 1.13) | 0.78 (0.59, 1.02) | 0.74 (0.55, 0.99) | 0.02 |
| **Zinc** | | | | | |
| Range | (0.02, 5.8) | (5.8, 8.56) | (8.56, 12.75) | (12.75, 279.36) |  |
| Model 1 | 1.00 | 0.97 (0.74, 1.27) | 0.88 (0.69, 1.12) | 0.74 (0.59, 0.94) | 0.01 |
| Model 2 | 1.00 | 0.89 (0.68, 1.17) | 0.82 (0.63, 1.06) | 0.80 (0.60, 1.06) | 0.08 |
| Model 3 | 1.00 | 0.87 (0.67, 1.14) | 0.80 (0.62, 1.05) | 0.79 (0.60, 1.04) | 0.08 |
| **Copper** | | | | | |
| Range | (0.017, 0.688) | (0.688, 0.987) | (0.987, 1.366) | (1.366, 27.364) |  |
| Model 1 | 1.00 | 1.03 (0.85, 1.24) | 0.75 (0.60, 0.94) | 0.64 (0.52, 0.79) | < 0.0001 |
| Model 2 | 1.00 | 0.90 (0.75, 1.09) | 0.66 (0.53, 0.82) | 0.61 (0.47, 0.78) | < 0.0001 |
| Model 3 | 1.00 | 0.89 (0.74, 1.07) | 0.63 (0.50, 0.80) | 0.60 (0.46, 0.78) | < 0.0001 |
| **Selenium** | | | | | |
| Range | (0, 59.2) | (59.2, 86.1) | (86.1, 122.1) | (122.1, 593.1) |  |
| Model 1 | 1.00 | 0.93 (0.75, 1.15) | 0.88 (0.71, 1.09) | 0.67 (0.56, 0.81) | < 0.001 |
| Model 2 | 1.00 | 1.01 (0.82, 1.25) | 0.99 (0.78, 1.25) | 0.93 (0.71, 1.20) | 0.58 |
| Model 3 | 1.00 | 0.99 (0.80, 1.22) | 0.97 (0.76, 1.23) | 0.92 (0.71, 1.20) | 0.56 |

Abbreviations: HR, hazard ratio; CI, confidence interval; ASCVD, atherosclerotic cardiovascular diseases;

Data are presented as HR (95% CI) unless indicated otherwise.

Model 1: crude model.

Model 2: adjusted for age, sex, race/ethnicity, education, smoking status, drinking status, leisure-time physical activity, body mass index, and energy intake.

Model 3: adjusted for age, sex, race/ethnicity, education, smoking status, drinking status, leisure-time physical activity, body mass index, energy intake, total cholesterol, high density lipoprotein, antihypertensive drug, antihyperlipidemic drug, diabetes, hypertension, and hyperlipidemia.

## **Supplementary Table S4**. HR (95% CIs) of all-cause mortality according to quintiles of dietary mineral intake among individuals with ASCVD.

| Characteristics | Quintiles of dietary minerals intake | | | | | *P* for trend |
| --- | --- | --- | --- | --- | --- | --- |
|  | Quintile 1 | Quintile 2 | Quintile 3 | Quintile 4 | Quintile 5 |  |
| **Calcium** | | | | | | |
| Range | (10, 392) | (392, 588.6) | (588.6, 811) | (811, 1,124.6) | (1,124.6, 6,408) |  |
| Model 1 | 1.00 | 0.93 (0.76, 1.16) | 0.81 (0.66, 0.99) | 0.90 (0.74, 1.10) | 0.76 (0.60, 0.95) | 0.02 |
| Model 2 | 1.00 | 0.86 (0.69, 1.09) | 0.82 (0.66, 1.01) | 0.95 (0.75, 1.21) | 0.89 (0.67, 1.17) | 0.66 |
| Model 3 | 1.00 | 0.86 (0.68, 1.10) | 0.81 (0.65, 1.01) | 0.96 (0.76, 1.21) | 0.87 (0.67, 1.15) | 0.6 |
| **Iron** | | | | | | |
| Range | (0.02, 7.53) | (7.53, 10.46) | (10.46, 13.584) | (13.584, 18.67) | (18.67, 111.04) |  |
| Model 1 | 1.00 | 1.03 (0.81, 1.31) | 0.89 (0.72, 1.10) | 1.00 (0.82, 1.23) | 0.86 (0.68, 1.09) | 0.22 |
| Model 2 | 1.00 | 0.95 (0.76, 1.20) | 0.84 (0.68, 1.04) | 0.94 (0.73, 1.20) | 0.81 (0.62, 1.06) | 0.21 |
| Model 3 | 1.00 | 0.93 (0.74, 1.17) | 0.83 (0.67, 1.03) | 0.95 (0.74, 1.22) | 0.79 (0.60, 1.04) | 0.19 |
| **Magnesium** | | | | | | |
| Range | (4, 152) | (152, 207) | (207, 267) | (267, 346) | (346, 1,704) |  |
| Model 1 | 1.00 | 0.97 (0.80, 1.18) | 0.90 (0.73, 1.10) | 0.76 (0.62, 0.93) | 0.59 (0.48, 0.74) | < 0.0001 |
| Model 2 | 1.00 | 0.82 (0.68, 0.99) | 0.81 (0.64, 1.01) | 0.70 (0.55, 0.89) | 0.58 (0.44, 0.78) | < 0.001 |
| Model 3 | 1.00 | 0.82 (0.67, 1.00) | 0.81 (0.63, 1.03) | 0.68 (0.52, 0.87) | 0.59 (0.43, 0.79) | < 0.001 |
| **Phosphorus** | | | | | | |
| Range | (0, 691.8) | (691.8, 933.6) | (933.6, 1,200) | (1,200, 1,564.2) | (1,564.2, 6,062) |  |
| Model 1 | 1.00 | 0.80 (0.64, 1.01) | 0.75 (0.61, 0.91) | 0.71 (0.57, 0.89) | 0.64 (0.51, 0.79) | < 0.0001 |
| Model 2 | 1.00 | 0.86 (0.70, 1.06) | 0.79 (0.63, 0.99) | 0.81 (0.62, 1.08) | 0.82 (0.59, 1.12) | 0.18 |
| Model 3 | 1.00 | 0.84 (0.69, 1.04) | 0.76 (0.60, 0.95) | 0.80 (0.61, 1.06) | 0.76 (0.55, 1.05) | 0.1 |
| **Potassium** | | | | | | |
| Range | (9, 1,440) | (1,440, 2,008) | (2,008, 2,543) | (2,543, 3,268.2) | (3,268.2, 11,533) |  |
| Model 1 | 1.00 | 0.85 (0.70, 1.05) | 0.93 (0.74, 1.17) | 0.79 (0.65, 0.95) | 0.64 (0.51, 0.80) | < 0.0001 |
| Model 2 | 1.00 | 0.73 (0.60, 0.88) | 0.78 (0.62, 0.98) | 0.64 (0.51, 0.80) | 0.62 (0.48, 0.82) | 0.002 |
| Model 3 | 1.00 | 0.72 (0.59, 0.88) | 0.77 (0.61, 0.98) | 0.61 (0.48, 0.77) | 0.59 (0.45, 0.78) | < 0.001 |
| **Sodium** | | | | | | |
| Range | (5, 1,731) | (1,731, 2,379) | (2,379, 3,084.4) | (3,084.4, 4,075.8) | (4,075.8, 18,053) |  |
| Model 1 | 1.00 | 0.86 (0.70, 1.05) | 0.78 (0.62, 0.98) | 0.66 (0.51, 0.86) | 0.54 (0.43, 0.67) | < 0.0001 |
| Model 2 | 1.00 | 0.90 (0.73, 1.11) | 0.77 (0.61, 0.98) | 0.75 (0.56, 1.01) | 0.68 (0.50, 0.93) | 0.01 |
| Model 3 | 1.00 | 0.83 (0.68, 1.02) | 0.72 (0.57, 0.90) | 0.70 (0.53, 0.93) | 0.62 (0.46, 0.82) | 0.002 |
| **Zinc** | | | | | | |
| Range | (0.02, 5.25) | (5.25, 7.516) | (7.516, 9.934) | (9.934, 13.992) | (13.992, 279.36) |  |
| Model 1 | 1.00 | 1.09 (0.86, 1.38) | 0.80 (0.62, 1.02) | 0.80 (0.63, 1.01) | 0.77 (0.61, 0.96) | 0.001 |
| Model 2 | 1.00 | 1.03 (0.82, 1.31) | 0.80 (0.63, 1.02) | 0.79 (0.61, 1.02) | 0.87 (0.66, 1.14) | 0.06 |
| Model 3 | 1.00 | 1.03 (0.82, 1.29) | 0.78 (0.61, 0.99) | 0.77 (0.60, 1.01) | 0.86 (0.66, 1.11) | 0.04 |
| **Copper** | | | | | | |
| Range | (0.017, 0.633) | (0.633, 0.867) | (0.867, 1.119) | (1.119, 1.471) | (1.471, 27.634) |  |
| Model 1 | 1.00 | 1.04 (0.87, 1.24) | 0.95 (0.77, 1.17) | 0.68 (0.54, 0.85) | 0.67 (0.54, 0.83) | < 0.0001 |
| Model 2 | 1.00 | 0.92 (0.77, 1.11) | 0.84 (0.69, 1.01) | 0.63 (0.49, 0.82) | 0.65 (0.50, 0.86) | < 0.001 |
| Model 3 | 1.00 | 0.89 (0.74, 1.07) | 0.80 (0.66, 0.98) | 0.60 (0.46, 0.80) | 0.64 (0.48, 0.85) | < 0.001 |
| **Selenium** | | | | | | |
| Range | (0, 54) | (54, 75.5) | (75.5, 98.2) | (98.2, 132.42) | (132.42, 593.1) |  |
| Model 1 | 1.00 | 0.87 (0.70, 1.06) | 0.71 (0.57, 0.88) | 0.73 (0.59, 0.90) | 0.58 (0.47, 0.71) | < 0.0001 |
| Model 2 | 1.00 | 0.92 (0.75, 1.14) | 0.74 (0.59, 0.94) | 0.87 (0.69, 1.11) | 0.77 (0.59, 1.00) | 0.05 |
| Model 3 | 1.00 | 0.93 (0.76, 1.13) | 0.72 (0.57, 0.89) | 0.88 (0.69, 1.13) | 0.77 (0.59, 1.01) | 0.05 |

Abbreviations: HR, hazard ratio; CI, confidence interval; ASCVD, atherosclerotic cardiovascular diseases;

Data are presented as HR (95% CI) unless indicated otherwise.

Model 1: crude model.

Model 2: adjusted for age, sex, race/ethnicity, education, smoking status, drinking status, leisure-time physical activity, body mass index, and energy intake.

Model 3: adjusted for age, sex, race/ethnicity, education, smoking status, drinking status, leisure-time physical activity, body mass index, energy intake, total cholesterol, high density lipoprotein, antihypertensive drug, antihyperlipidemic drug, diabetes, hypertension, and hyperlipidemia.

## **Supplementary Table S5**. HRs (95% CIs) of all-cause mortality according to dietary minerals intake among ASCVD individuals with further adjustment of several liver function biomarkers.

| Characteristics | Quartiles of dietary minerals intake | | | | *P* for trend |
| --- | --- | --- | --- | --- | --- |
|  | Quartile 1 | Quartile 2 | Quartile 3 | Quartile 4 |  |
| **Calcium** | | | | | |
| Range | (10, 441) | (441, 691) | (691, 1,033) | (1,033, 6,408) |  |
| Model 1 | 1.00 | 0.88 (0.72, 1.07) | 0.88 (0.75, 1.04) | 0.73 (0.59, 0.89) | 0.002 |
| Model 2 | 1.00 | 0.86 (0.71, 1.04) | 0.95 (0.80, 1.14) | 0.84 (0.66, 1.07) | 0.31 |
| Model 3 | 1.00 | 0.86 (0.71, 1.04) | 0.96 (0.80, 1.16) | 0.84 (0.66, 1.06) | 0.28 |
| Model 4 | 1.00 | 0.86 (0.71, 1.05) | 0.97 (0.80, 1.17) | 0.84 (0.66, 1.06) | 0.29 |
| **Iron** | | | | | |
| Range | (0.02, 8.29) | (8.29, 11.94) | (11.94, 17.16) | (17.16, 111.04) |  |
| Model 1 | 1.00 | 0.89 (0.72, 1.10) | 0.94 (0.77, 1.15) | 0.81 (0.64, 1.01) | 0.12 |
| Model 2 | 1.00 | 0.78 (0.64, 0.97) | 0.84 (0.66, 1.06) | 0.70 (0.54, 0.91) | 0.03 |
| Model 3 | 1.00 | 0.77 (0.63, 0.95) | 0.82 (0.65, 1.03) | 0.68 (0.52, 0.88) | 0.02 |
| Model 4 | 1.00 | 0.77 (0.63, 0.95) | 0.82 (0.65, 1.03) | 0.68 (0.52, 0.88) | 0.02 |
| **Magnesium** | | | | | |
| Range | (4, 166) | (166, 236) | (236, 324) | (324, 1704) |  |
| Model 1 | 1.00 | 0.94 (0.81, 1.10) | 0.77 (0.64, 0.93) | 0.61 (0.50, 0.74) | < 0.0001 |
| Model 2 | 1.00 | 0.87 (0.74, 1.01) | 0.72 (0.59, 0.89) | 0.61 (0.48, 0.79) | < 0.001 |
| Model 3 | 1.00 | 0.85 (0.72, 1.01) | 0.71 (0.57, 0.88) | 0.60 (0.46, 0.77) | < 0.0001 |
| Model 4 | 1.00 | 0.86 (0.73, 1.01) | 0.72 (0.58, 0.89) | 0.60 (0.46, 0.78) | < 0.0001 |
| **Phosphorus** | | | | | |
| Range | (1, 757) | (757, 1,069) | (1,069, 1,455) | (1,455, 6,062) |  |
| Model 1 | 1.00 | 0.90 (0.75, 1.08) | 0.86 (0.69, 1.05) | 0.69 (0.57, 0.82) | < 0.0001 |
| Model 2 | 1.00 | 0.98 (0.82, 1.19) | 0.90 (0.71, 1.15) | 0.82 (0.62, 1.10) | 0.18 |
| Model 3 | 1.00 | 0.97 (0.80, 1.16) | 0.86 (0.67, 1.10) | 0.77 (0.58, 1.03) | 0.07 |
| Model 4 | 1.00 | 0.96 (0.80, 1.16) | 0.86 (0.67, 1.11) | 0.77 (0.58, 1.03) | 0.08 |
| **Potassium** | | | | | |
| Range | (29, 1,585) | (1,585, 2,278) | (2,278, 3,066) | (3,066, 11,533) |  |
| Model 1 | 1.00 | 0.94 (0.76, 1.15) | 0.95 (0.78, 1.16) | 0.68 (0.54, 0.85) | < 0.001 |
| Model 2 | 1.00 | 0.81 (0.68, 0.96) | 0.83 (0.66, 1.03) | 0.69 (0.53, 0.89) | 0.01 |
| Model 3 | 1.00 | 0.81 (0.68, 0.96) | 0.79 (0.63, 1.00) | 0.64 (0.50, 0.83) | 0.003 |
| Model 4 | 1.00 | 0.81 (0.68, 0.96) | 0.80 (0.63, 1.01) | 0.65 (0.50, 0.84) | 0.004 |
| **Sodium** | | | | | |
| Range | (5, 1,914) | (1,914, 2,714) | (2,714, 3,780) | (3,780, 18,053) |  |
| Model 1 | 1.00 | 0.75 (0.61, 0.92) | 0.66 (0.51, 0.85) | 0.55 (0.44, 0.69) | < 0.0001 |
| Model 2 | 1.00 | 0.78 (0.63, 0.95) | 0.72 (0.54, 0.96) | 0.70 (0.51, 0.95) | 0.02 |
| Model 3 | 1.00 | 0.75 (0.62, 0.90) | 0.68 (0.51, 0.90) | 0.65 (0.48, 0.86) | 0.003 |
| Model 4 | 1.00 | 0.74 (0.61, 0.89) | 0.68 (0.51, 0.90) | 0.65 (0.48, 0.87) | 0.003 |
| **Zinc** | | | | | |
| Range | (0.02, 5.79) | (5.79, 8.59) | (8.59, 12.79) | (12.79, 279.36) |  |
| Model 1 | 1.00 | 0.92 (0.72, 1.17) | 0.83 (0.66, 1.04) | 0.73 (0.58, 0.92) | 0.003 |
| Model 2 | 1.00 | 0.85 (0.67, 1.07) | 0.77 (0.61, 0.97) | 0.79 (0.60, 1.03) | 0.06 |
| Model 3 | 1.00 | 0.83 (0.66, 1.04) | 0.76 (0.60, 0.95) | 0.77 (0.59, 0.99) | 0.03 |
| Model 4 | 1.00 | 0.83 (0.67, 1.04) | 0.76 (0.60, 0.97) | 0.77 (0.60, 1.01) | 0.05 |
| **Copper** | | | | | |
| Range | (0.017, 0.687) | (0.687, 0.991) | (0.991, 1.365) | (1.365, 27.364) |  |
| Model 1 | 1.00 | 1.02 (0.86, 1.22) | 0.75 (0.61, 0.91) | 0.65 (0.53, 0.79) | < 0.0001 |
| Model 2 | 1.00 | 0.89 (0.75, 1.05) | 0.66 (0.53, 0.84) | 0.63 (0.48, 0.81) | < 0.001 |
| Model 3 | 1.00 | 0.88 (0.74, 1.04) | 0.63 (0.50, 0.81) | 0.61 (0.47, 0.80) | < 0.0001 |
| Model 4 | 1.00 | 0.88 (0.74, 1.04) | 0.63 (0.50, 0.80) | 0.62 (0.47, 0.80) | < 0.0001 |
| **Selenium** | | | | | |
| Range | (0, 59.3) | (59.3, 86.1) | (86.1, 122) | (122, 593.1) |  |
| Model 1 | 1.00 | 0.82 (0.67, 1.00) | 0.77 (0.64, 0.93) | 0.63 (0.53, 0.74) | < 0.0001 |
| Model 2 | 1.00 | 0.86 (0.70, 1.04) | 0.84 (0.68, 1.03) | 0.83 (0.66, 1.03) | 0.08 |
| Model 3 | 1.00 | 0.84 (0.69, 1.01) | 0.82 (0.66, 1.01) | 0.81 (0.65, 1.01) | 0.06 |
| Model 4 | 1.00 | 0.83 (0.69, 1.01) | 0.82 (0.66, 1.01) | 0.81 (0.65, 1.02) | 0.06 |

Abbreviations: HR, hazard ratio; CI, confidence interval; ASCVD, atherosclerotic cardiovascular diseases;

Data are presented as HR (95% CI) unless indicated otherwise.

Model 1: crude model.

Model 2: adjusted for age, sex, race/ethnicity, education, smoking status, drinking status, leisure-time physical activity, body mass index, and energy intake.

Model 3: adjusted for age, sex, race/ethnicity, education, smoking status, drinking status, leisure-time physical activity, body mass index, energy intake, total cholesterol, high density lipoprotein, antihypertensive drug, antihyperlipidemic drug, diabetes, hypertension, and hyperlipidemia.

Model 4: adjusted for age, sex, race/ethnicity, education, smoking status, drinking status, leisure-time physical activity, body mass index, energy intake, total cholesterol, high density lipoprotein, antihypertensive drug, antihyperlipidemic drug, diabetes, hypertension, hyperlipidemia, alanine aminotransferase, and aspartate aminotransferase.

## **Supplementary Table S6**. Subgroup analyses for all-cause mortality according to dietary minerals intake after stratification by BMI

| Characteristics | Quartiles of dietary minerals intake | | | | *P* for trend | *P* for interaction |
| --- | --- | --- | --- | --- | --- | --- |
|  | Quartile 1 | Quartile 2 | Quartile 3 | Quartile 4 |  |  |
| **Calcium** | | | | | | |
| Range | (10, 439) | (439, 689) | (689, 1,033) | (1,033, 6,408) |  |  |
| BMI |  |  |  |  |  | 0.021 |
| < 25 | 1.000 | 0.952 (0.678, 1.338) | 0.837 (0.572, 1.225) | 0.912 (0.612, 1.359) | 0.494 |  |
| 25-30 | 1.000 | 0.866 (0.634, 1.184) | 0.893 (0.664, 1.202) | 0.699 (0.513, 0.952) | 0.041 |  |
| ≥ 30 | 1.000 | 0.873 (0.650, 1.172) | 1.012 (0.716, 1.430) | 1.022 (0.675, 1.550) | 0.722 |  |
| **Iron** | | | | | | |
| Range | (0.02, 8.27) | (8.27, 11.92) | (11.92, 17.14) | (17.14, 111.04) |  |  |
| BMI |  |  |  |  |  | 0.001 |
| < 25 | 1.000 | 0.588 (0.420, 0.824) | 0.608 (0.389, 0.952) | 0.463 (0.276, 0.779) | 0.008 |  |
| 25-30 | 1.000 | 0.860 (0.634, 1.166) | 0.906 (0.649, 1.265) | 0.679 (0.492, 0.938) | 0.029 |  |
| ≥ 30 | 1.000 | 0.931 (0.661, 1.313) | 0.872 (0.552, 1.377) | 0.910 (0.581, 1.426) | 0.67 |  |
| **Magnesium** | | | | | | |
| Range | (4, 166) | (166, 235) | (235, 324) | (324, 1,704) |  |  |
| BMI |  |  |  |  |  | < 0.0001 |
| < 25 | 1.000 | 0.990 (0.704, 1.393) | 0.578 (0.383, 0.874) | 0.487 (0.310, 0.764) | < 0.001 |  |
| 25-30 | 1.000 | 0.692 (0.547, 0.875) | 0.612 (0.456, 0.821) | 0.463 (0.322, 0.664) | < 0.0001 |  |
| ≥ 30 | 1.000 | 1.011 (0.713, 1.432) | 0.905 (0.596, 1.374) | 0.963 (0.580, 1.599) | 0.773 |  |
| **Phosphorus** | | | | | | |
| Range | (0, 752) | (752, 1,066) | (1,066, 1,454) | (1,454, 6,062) |  |  |
| BMI |  |  |  |  |  | < 0.001 |
| < 25 | 1.000 | 0.770 (0.552, 1.073) | 0.723 (0.506, 1.032) | 0.495 (0.308, 0.795) | 0.007 |  |
| 25-30 | 1.000 | 0.927 (0.705, 1.219) | 0.842 (0.585, 1.211) | 0.722 (0.473, 1.104) | 0.15 |  |
| ≥ 30 | 1.000 | 1.155 (0.807, 1.651) | 0.835 (0.519, 1.342) | 1.062 (0.589, 1.915) | 0.766 |  |
| **Potassium** | | | | | | |
| Range | (9, 1,583) | (1,583, 2,271) | (2,271, 3,058) | (3,058, 11,533) |  |  |
| BMI |  |  |  |  |  | < 0.0001 |
| < 25 | 1.000 | 0.776 (0.544, 1.107) | 0.642 (0.435, 0.949) | 0.379 (0.243, 0.591) | < 0.0001 |  |
| 25-30 | 1.000 | 0.624 (0.475, 0.820) | 0.610 (0.442, 0.843) | 0.554 (0.381, 0.807) | 0.005 |  |
| ≥ 30 | 1.000 | 1.064 (0.777, 1.457) | 0.992 (0.676, 1.457) | 1.029 (0.637, 1.660) | 0.997 |  |
| **Sodium** | | | | | | |
| Range | (5, 1,908) | (1,908, 2,708) | (2,708, 3,776) | (3,776, 18,053) |  |  |
| BMI |  |  |  |  |  | 0.009 |
| < 25 | 1.000 | 0.690 (0.507, 0.938) | 0.673 (0.452, 1.000) | 0.609 (0.383, 0.971) | 0.032 |  |
| 25-30 | 1.000 | 0.821 (0.621, 1.087) | 0.825 (0.588, 1.157) | 0.650 (0.429, 0.986) | 0.095 |  |
| ≥ 30 | 1.000 | 0.846 (0.626, 1.144) | 0.547 (0.372, 0.803) | 0.639 (0.405, 1.009) | 0.015 |  |
| **Zinc** | | | | | | |
| Range | (0.02, 5.8) | (5.8, 8.56) | (8.56, 12.75) | (12.75, 279.36) |  |  |
| BMI |  |  |  |  |  | 0.075 |
| < 25 | 1.000 | 0.816 (0.562, 1.185) | 0.554 (0.376, 0.817) | 0.700 (0.455, 1.076) | 0.044 |  |
| 25-30 | 1.000 | 1.013 (0.715, 1.434) | 0.868 (0.595, 1.265) | 0.850 (0.587, 1.230) | 0.265 |  |
| ≥ 30 | 1.000 | 0.787 (0.534, 1.159) | 0.788 (0.542, 1.147) | 0.736 (0.499, 1.085) | 0.184 |  |
| **Copper** | | | | | | |
| Range | (0.017, 0.688) | (0.688, 0.987) | (0.987, 1.366) | (1.366, 27.364) |  |  |
| BMI |  |  |  |  |  | < 0.001 |
| < 25 | 1.000 | 0.841 (0.624, 1.133) | 0.493 (0.332, 0.731) | 0.441 (0.293, 0.664) | < 0.0001 |  |
| 25-30 | 1.000 | 1.068 (0.835, 1.365) | 0.670 (0.495, 0.908) | 0.640 (0.457, 0.896) | 0.001 |  |
| ≥ 30 | 1.000 | 0.911 (0.651, 1.277) | 0.672 (0.407, 1.112) | 0.772 (0.474, 1.258) | 0.207 |  |
| **Selenium** | | | | | | |
| Range | (0, 59.2) | (59.2, 86.1) | (86.1, 122.1) | (122.1, 593.1) |  |  |
| BMI |  |  |  |  |  | 0.008 |
| < 25 | 1.000 | 0.544 (0.368, 0.803) | 0.582 (0.395, 0.857) | 0.550 (0.367, 0.826) | 0.011 |  |
| 25-30 | 1.000 | 1.080 (0.778, 1.499) | 0.935 (0.675, 1.296) | 0.959 (0.639, 1.438) | 0.674 |  |
| ≥ 30 | 1.000 | 0.888 (0.638, 1.237) | 0.802 (0.524, 1.226) | 0.778 (0.487, 1.243) | 0.278 |  |

Abbreviations: HR, hazard ratio; CI, confidence interval; BMI, body mass index.

Data are presented as HR (95% CI) unless indicated otherwise. Adjusted for age, sex, race/ethnicity, education, smoking status, drinking status, leisure-time physical activity, energy intake, total cholesterol, high density lipoprotein, antihypertensive drug, antihyperlipidemic drug, diabetes, hypertension, and hyperlipidemia.

# **Supplementary Figures**

## **Supplementary Figure S1**. The flow chart of participants inclusion and exclusion in this study.


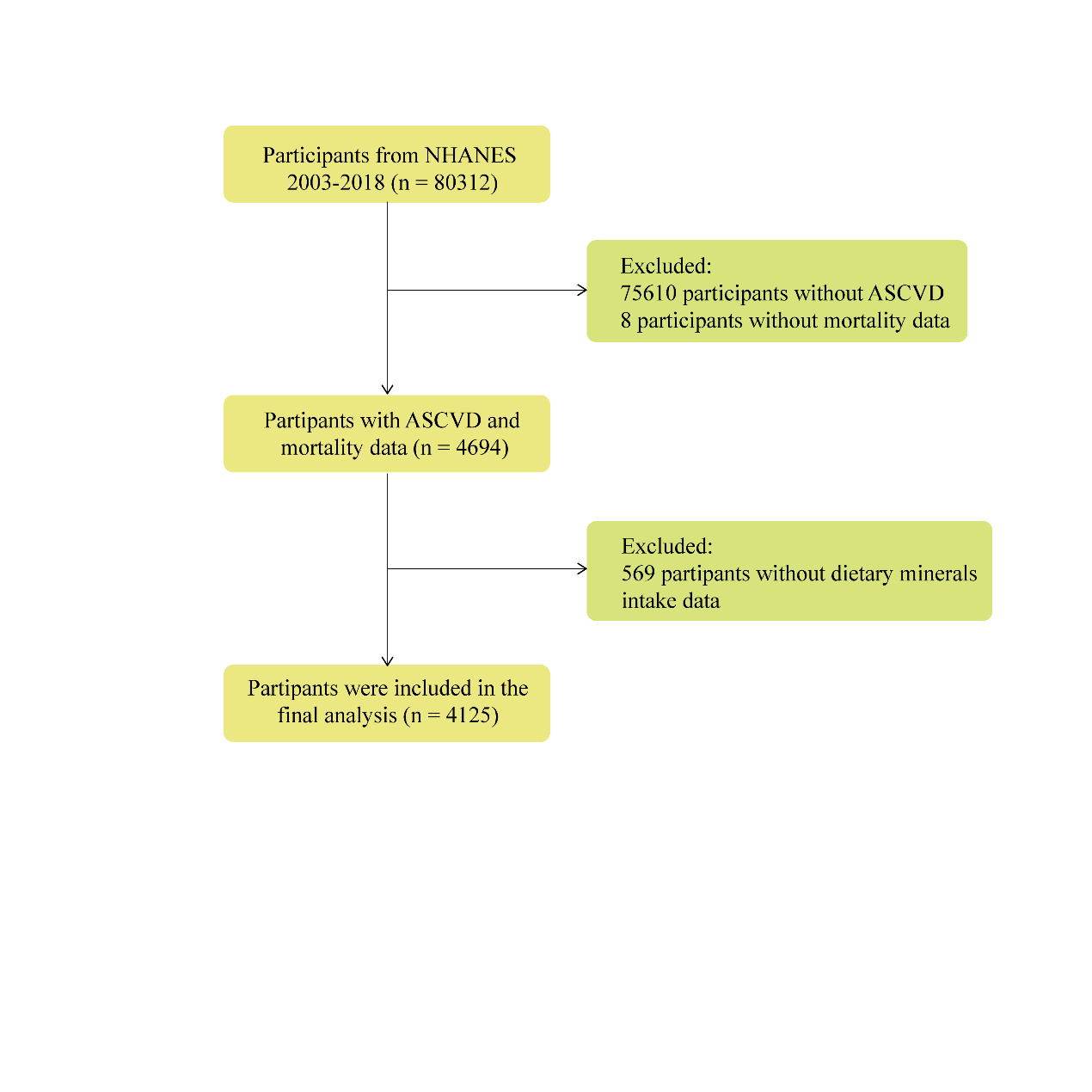


Abbreviations: NHANES, National Health and Nutrition Examination Survey; ASCVD, atherosclerotic cardiovascular diseases.

## **Supplementary Figure S2**. Kaplan-Meier survival analysis curves of dietary intakes of sodium (A), zinc (B), copper (C), and selenium (D) and all-cause mortality.


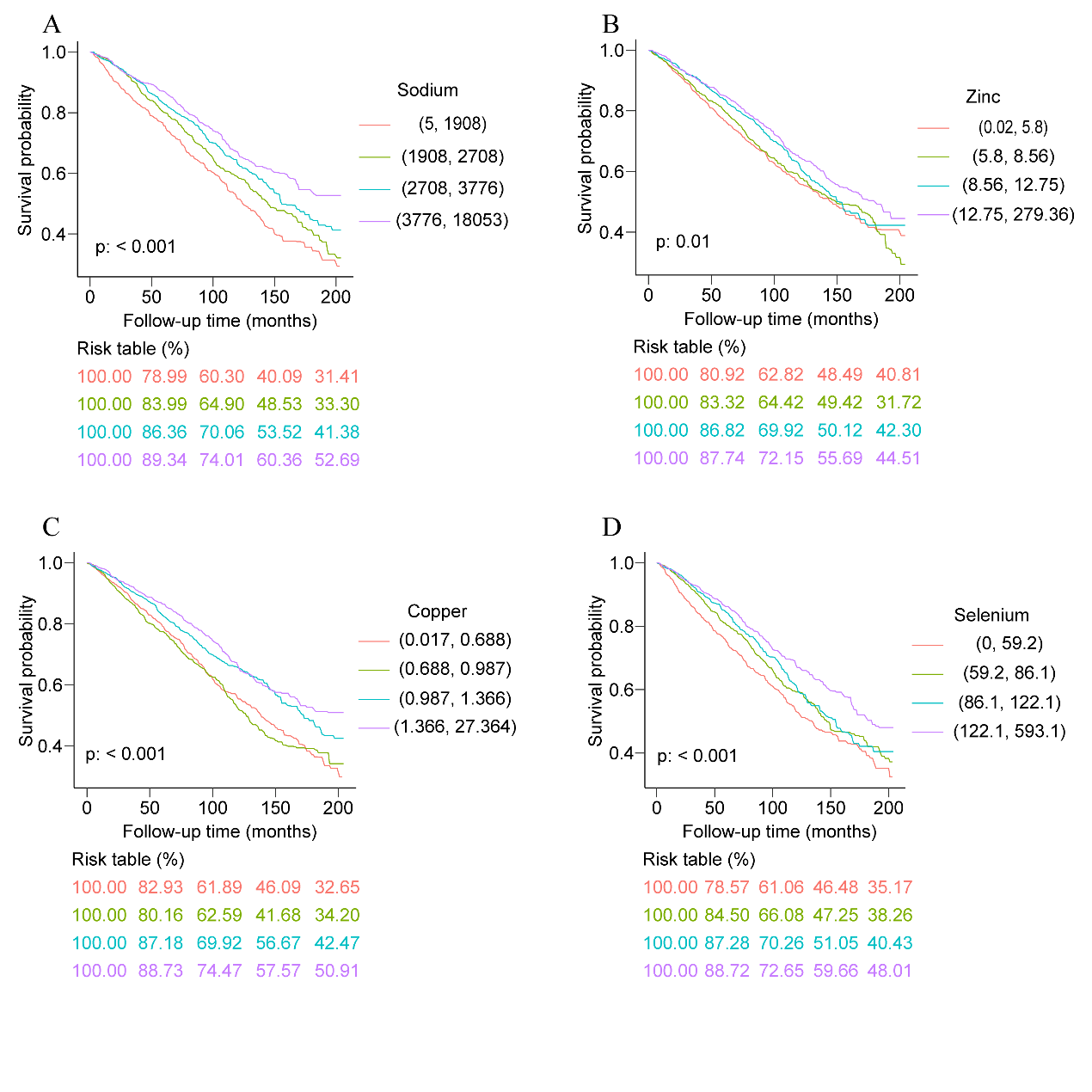


## **Supplementary Figure S3**. Kaplan-Meier survival analysis curve of dietary intake of iron and all-cause mortality.


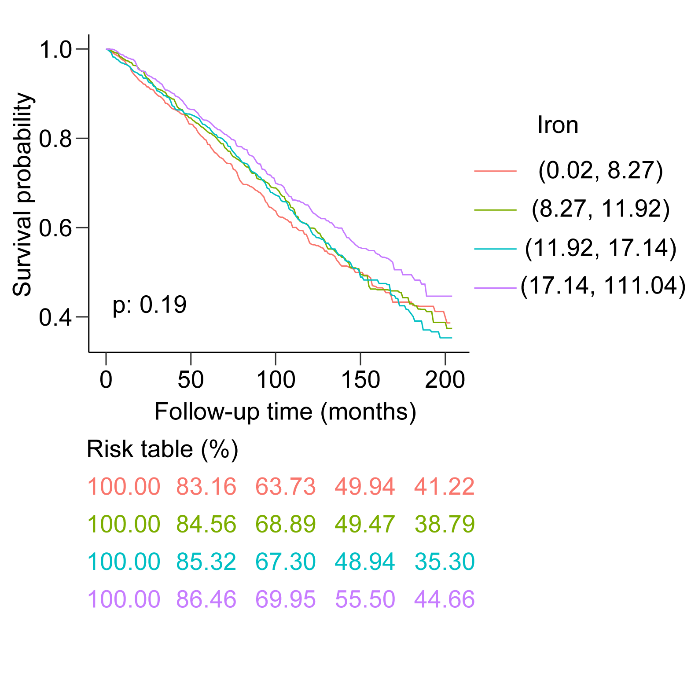


## **Supplementary Figure S4**. The relationships between dietary intakes of calcium (A), iron (B), phosphorus (C), and selenium (D), and all-cause mortality using restricted cubic spline method.


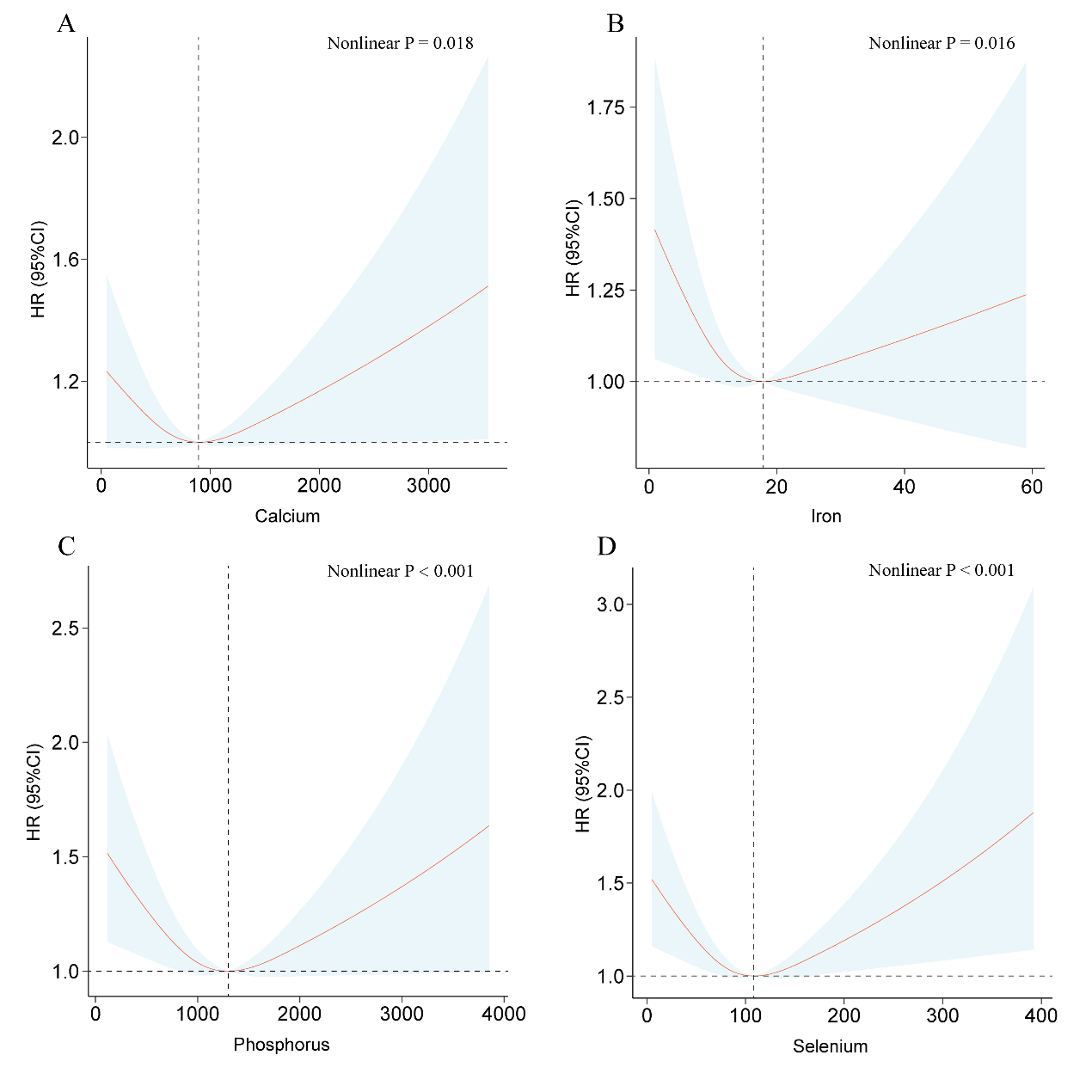


## **Supplementary Figure S5**. The relationships between dietary intakes of potassium (A), zinc (B), and copper (C), and all-cause mortality using restricted cubic spline method.


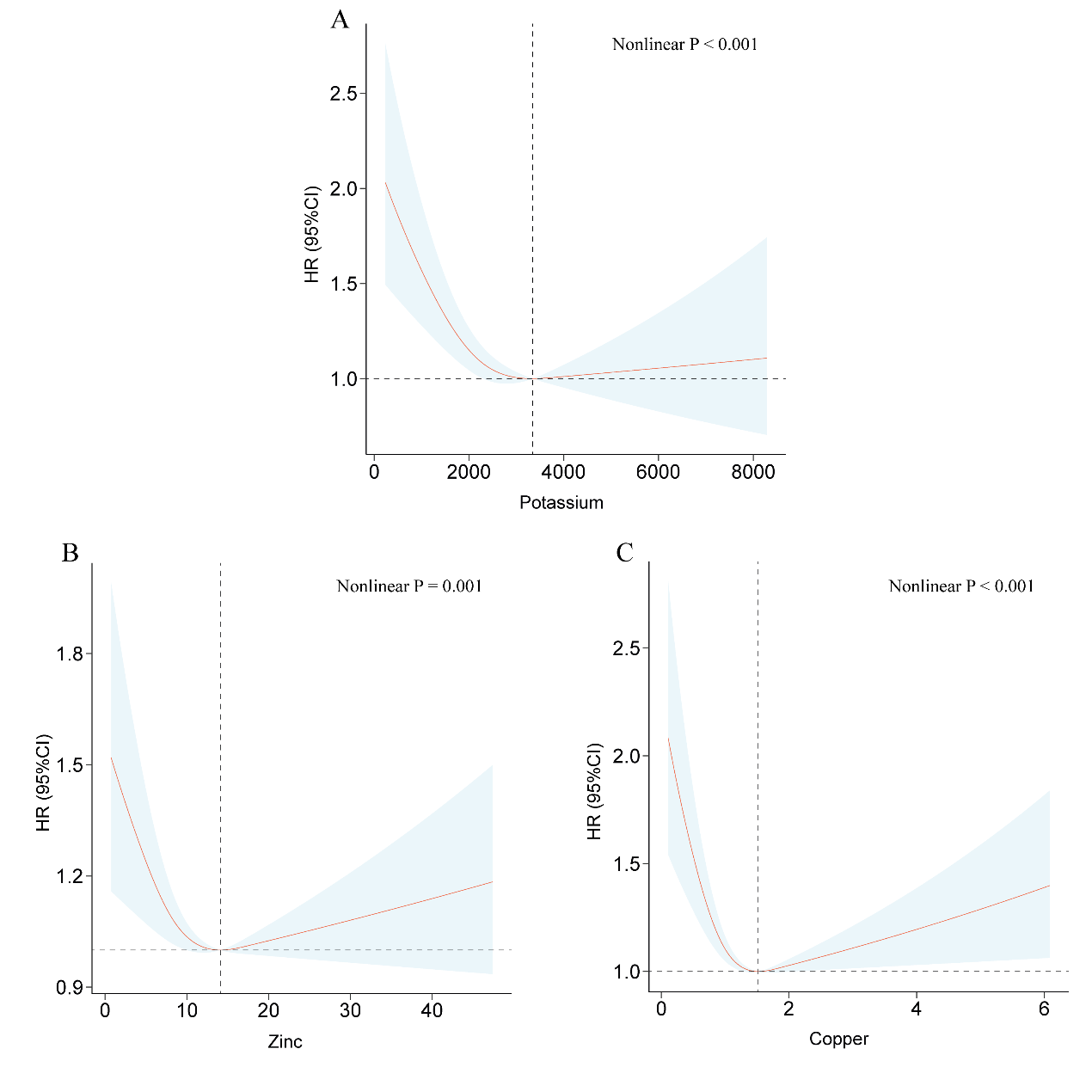


## **Supplementary Figure S6.** Kaplan-Meier survival analysis curves of dietary intakes of calcium (A, B, C), iron (D, E, F), and magnesium (G, H, I), and all-cause mortality after stratification by BMI.


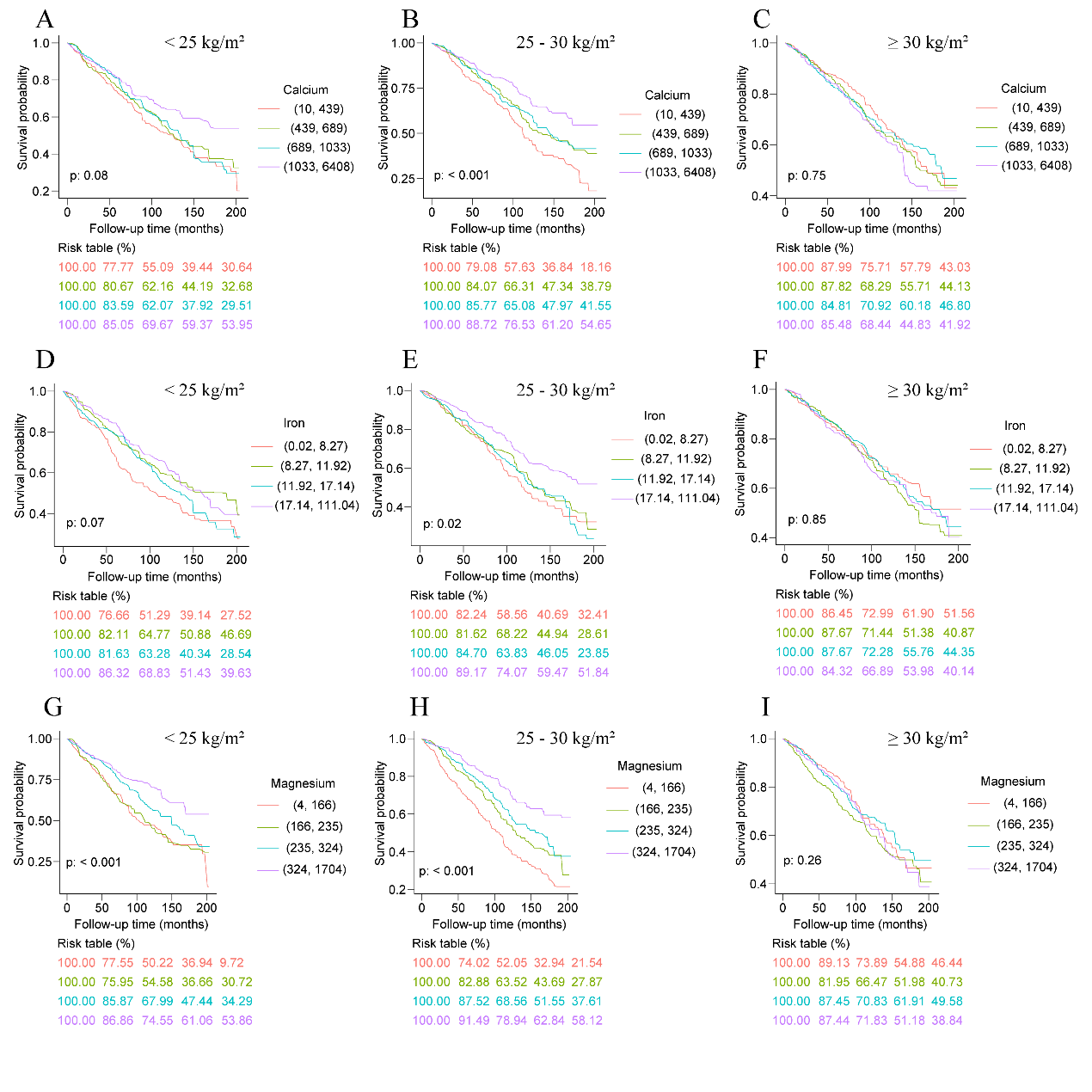


## **Supplementary Figure S7.** Kaplan-Meier survival analysis curves of dietary intakes of phosphorus (A, B, C), potassium (D, E, F), and sodium (G, H, I), and all-cause mortality after stratification by BMI.


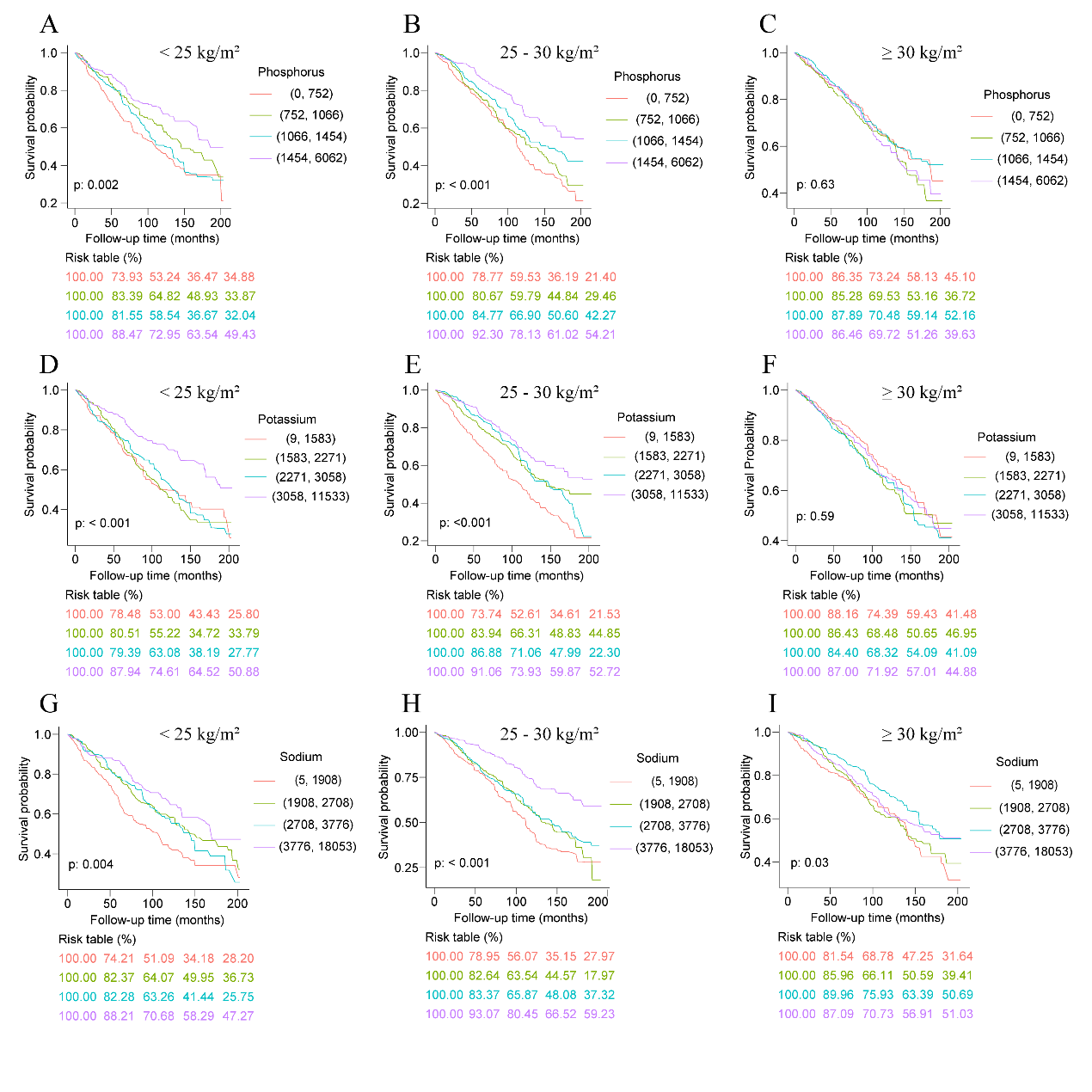


## **Supplementary Figure S8.** Kaplan-Meier survival analysis curves of dietary intakes of zinc (A, B, C), copper (D, E, F), and selenium (G, H, I), and all-cause mortality after stratification by BMI.


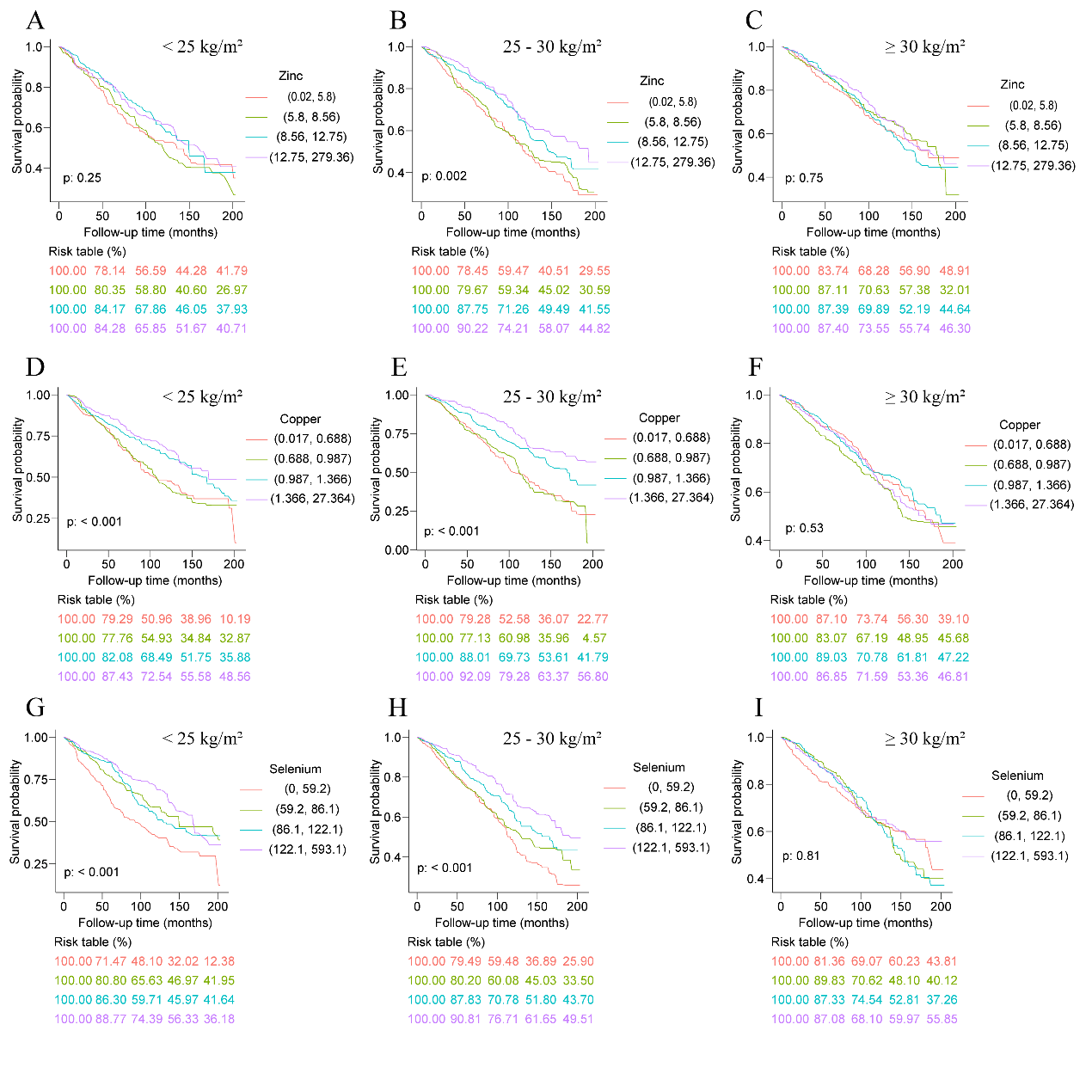


## **Supplementary Figure S9**. The associations between dietary intakes of calcium(A), iron (B), zinc (C), and selenium (D) and all-cause mortality within populations of normal or low body weight using restricted cubic spline method.


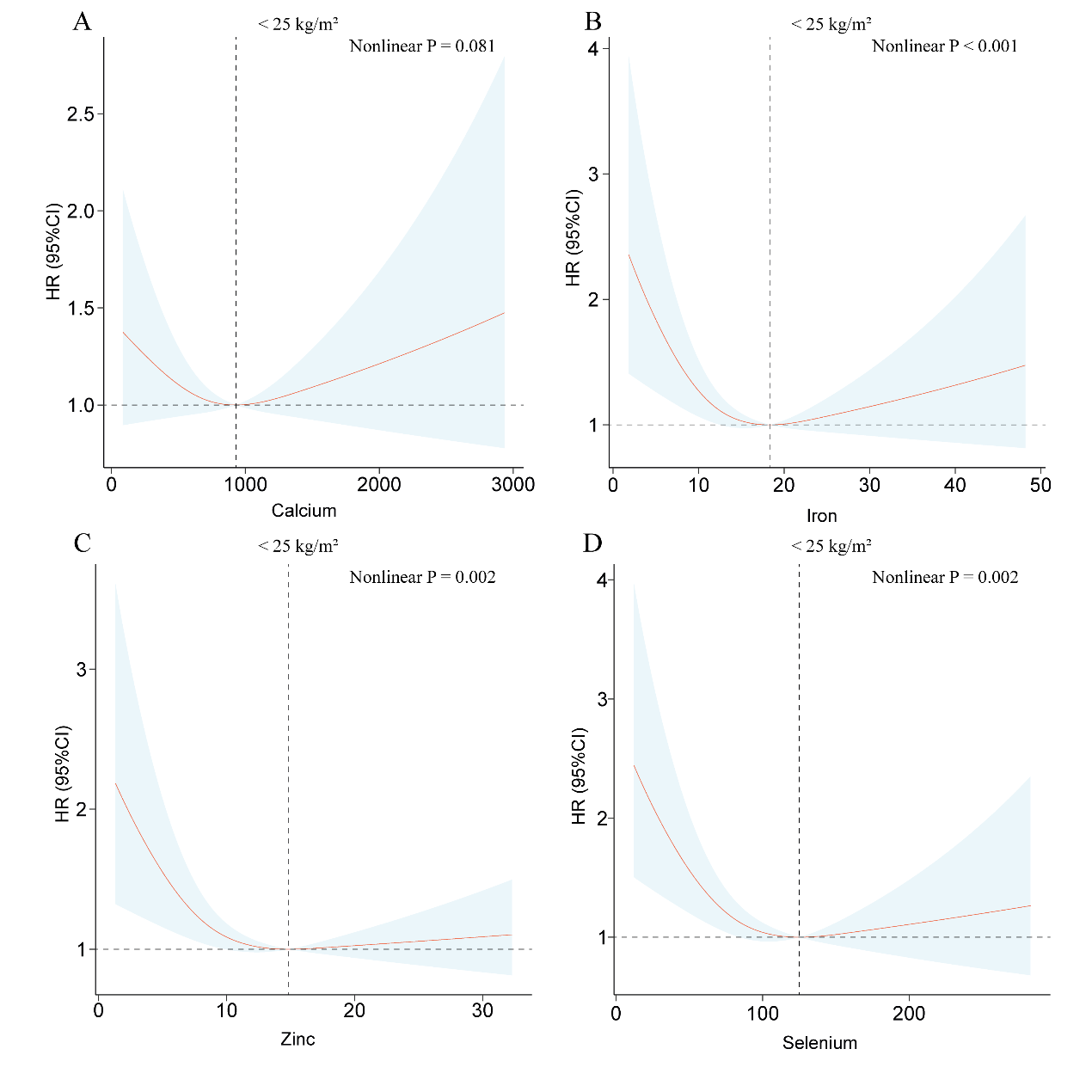


## **Supplementary Figure S10.** The associations between dietary intakes of calcium(A), iron (B), magnesium (C), phosphorus (D), potassium (E), and sodium (F) and all-cause mortality within populations of overweight using restricted cubic spline method.


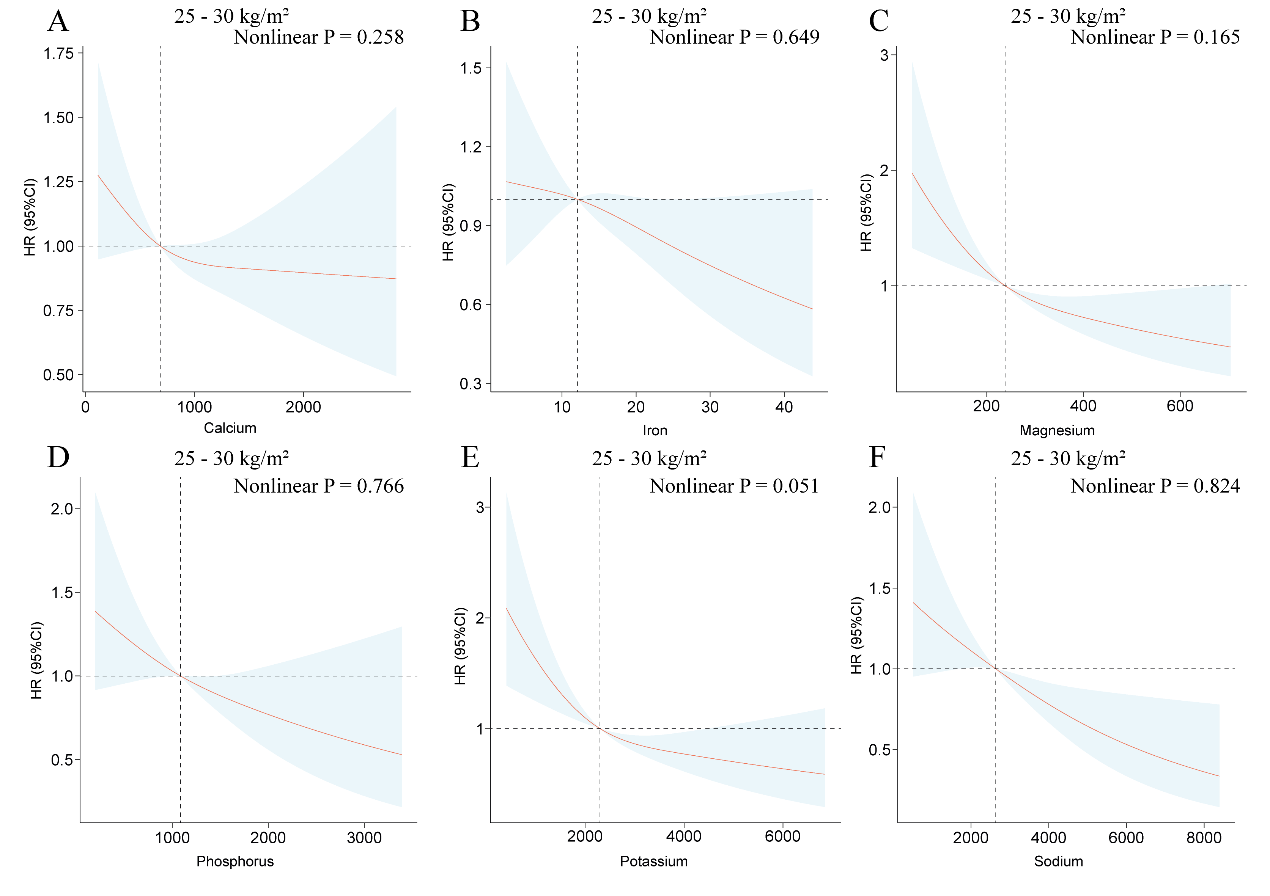


## **Supplementary Figure S11.** The associations between dietary intakes of zinc(A), copper (B), and selenium (C) and all-cause mortality within populations of overweight using restricted cubic spline method.


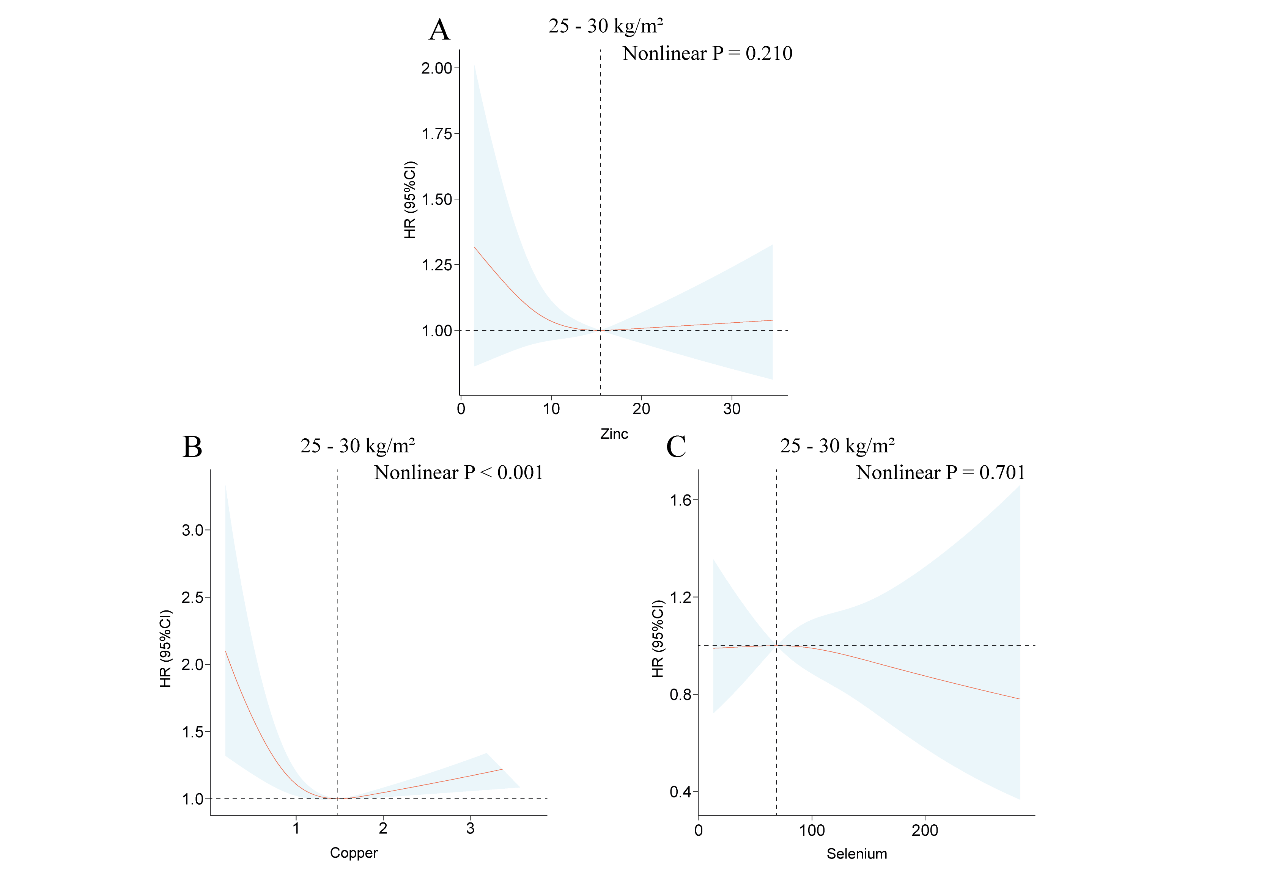


## **Supplementary Figure S12.** The associations between dietary intakes of calcium(A), iron (B), magnesium (C), phosphorus (D), potassium (E), sodium (F), zinc (G), copper (H), and selenium (I) and all-cause mortality within populations of obesity using restricted cubic spline method.


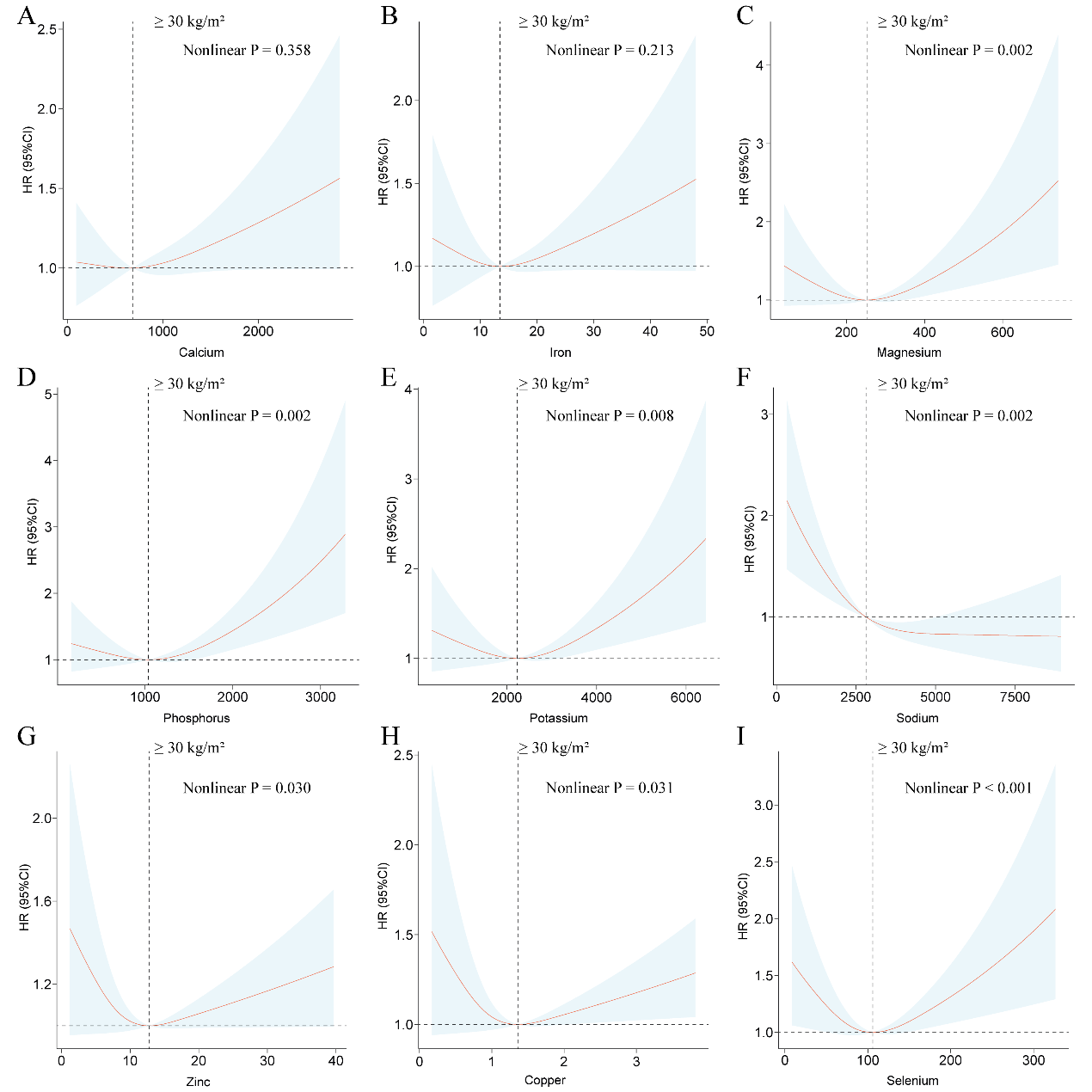


# **Supplementary method**

R software (version 4.3.0) was utilized for the execution of statistical analyses. Statistical significance was established at a two-tailed p-value threshold of 0.05. This study employed the "survey" package for weighted analysis and explored non-linear relationships using the "rms" package (1, 2).

**Reference**

1. T. Lumley. (2024). survey: analysis of complex survey samples. <http://r-survey.r-forge.r-project.org/survey/> [Accessed March 15, 2024].

2. Harrell Jr FE. 2024. rms: Regression Modeling Strategies. <https://hbiostat.org/R/rms/> [Accessed March 15, 2024].
